# Mitochondrial dysfunction in intracerebral hemorrhage: molecular mechanisms and pathological consequences

**DOI:** 10.3389/fncel.2026.1913984

**Published:** 2026-08-06

**Authors:** Tong Chen, Xu Gao, Yuhao Chang, Bin Wang, Zhouying Shi, Haiyue Xu, Yue Yuan, Fan Li, Ping Li

**Affiliations:** 1 College of Traditional Chinese Medicine, Changchun University of Chinese Medicine, Changchun, China; 2 College of Basic Medicine, Changchun University of Chinese Medicine, Changchun, China; 3 College of Nursing, Changchun University of Chinese Medicine, Changchun, China; 4 The First Affiliated Hospital to Changchun University of Chinese Medicine, Changchun, China

**Keywords:** cell death, intracerebral hemorrhage, mitochondrial dysfunction, mitochondrial quality control, mitochondrial reactive oxygen species, secondary brain injury, stroke

## Abstract

Intracerebral hemorrhage (ICH) carries high rates of disability and mortality, with a poor prognosis largely attributable to secondary brain injury (SBI). Mitochondria, a central hub for cellular redox regulation and energy metabolism, are not only affected by SBI but can also, once dysfunctional, initiate and exacerbate SBI. In this review, we classify post-ICH mitochondrial dysfunction into three major categories: excessive accumulation of mitochondrial reactive oxygen species, dysregulation of energy metabolism, and dysregulation of mitochondrial quantity and quality control. We delineate the molecular mechanisms and pathological consequences of each. Furthermore, given the extensive crosstalk between mitochondrial dysfunction and diverse forms of cell death in ICH (e.g., apoptosis, necrosis, necroptosis, ferroptosis, and pyroptosis), we discuss their mechanistic links. Overall, this review proposes a mitochondria-centered framework and emphasizes crosstalk between different types of dysfunction, thereby advancing our understanding of SBI pathophysiology after ICH and providing a systematic perspective on therapeutic strategies aimed at restoring mitochondrial homeostasis.

## 1 Introduction

Intracerebral hemorrhage (ICH), defined as spontaneous nontraumatic bleeding within the brain parenchyma, is a severe and fatal type of stroke, accounting for 28.8% of all stroke cases worldwide (Parry-Jones et al., 2025). Its mortality and disability rates are extremely high: the 1-month case fatality rate is approximately 35.5%, and only about 31.2% of survivors achieve a favorable functional outcome (Parry-Jones et al., 2025; van Asch et al., 2010; Cordonnier et al., 2018). Despite this substantial disease burden, effective treatments for ICH remain limited (Seiffge et al., 2024). Understanding the mechanisms of ICH-induced brain injury is therefore central to the identification of therapeutic targets.

**FIGURE 1:**
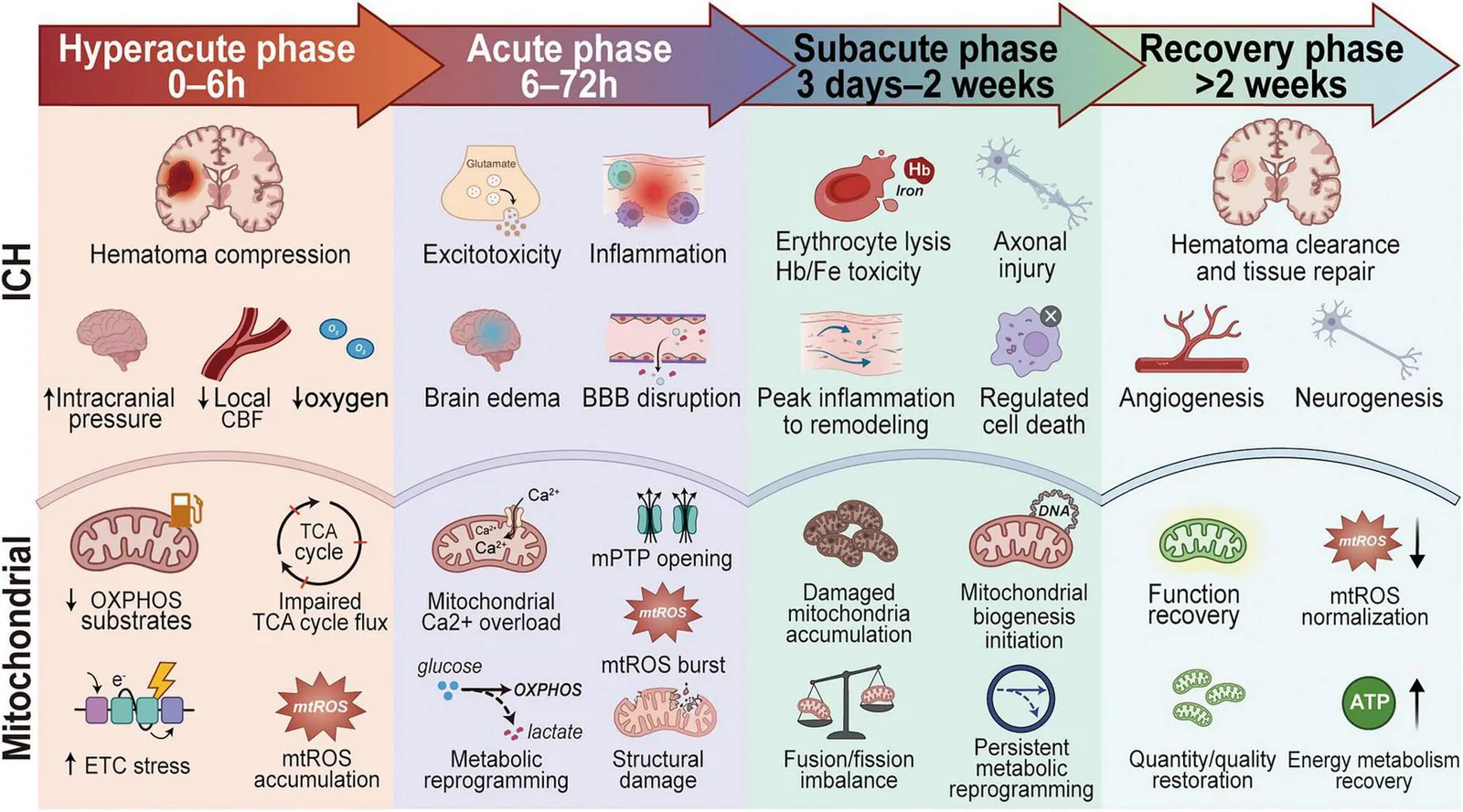
Temporal evolution of pathological processes and mitochondrial dysfunction after ICH. After ICH, a complex series of pathophysiological changes evolves over hours to weeks and contributes to brain injury, with mitochondrial dysfunction accompanying the entire injury course. The predominant mechanisms of brain injury vary across different stages, and the mechanisms of mitochondrial dysfunction likewise exhibit time-dependent features.

Following ICH, the mechanical compression and mass effect of the hematoma directly damage brain tissue, termed primary injury (Keep et al., 2012). However, secondary brain injury (SBI) is the principal driver of neurological deterioration and poor prognosis in ICH patients. SBI encompasses a complex cascade of molecular and cellular responses that unfold within hours to days after the initial hemorrhage, including the toxic effects of hematoma components, local neuroinflammation, oxidative stress, excitotoxicity, blood–brain barrier (BBB) disruption, brain edema, and mitochondrial dysfunction (Figure 1; Kim-Han et al., 2006; Lee et al., 2006; Aronowski and Zhao, 2011; Keep et al., 2012). Notably, mitochondrial dysfunction is not simply a consequence of injury but an active driver of SBI. This central role stems from the brain’s unique metabolic characteristics: the brain consumes approximately 20% of the body’s total energy and is highly dependent on mitochondrial oxidative phosphorylation (Magistretti and Allaman, 2015); neurons possess limited glycolytic capacity and are highly vulnerable to energy deficits (Herrero-Mendez et al., 2009; Magistretti and Allaman, 2015). Moreover, as the primary source of reactive oxygen species (ROS), mitochondria play a critical role in cellular redox regulation (Balaban et al., 2005).

Existing research articles and reviews on ICH and mitochondrial dysfunction have largely focused on specific mitochondrial processes, such as mitochondrial reactive oxygen species (mtROS) accumulation, mitochondrial dynamics, mitophagy, or mitochondrial quality control (Xiao et al., 2021; Zheng et al., 2023; Chen et al., 2024; Liu M. et al., 2025; Shang et al., 2025; Tang et al., 2025). These studies have greatly advanced our understanding of the mechanisms underlying mitochondrial abnormalities after ICH; however, they have rarely undertaken a systematic integration of the interconnections among different mitochondrial abnormalities and their collective roles in secondary brain injury from the perspective of global mitochondrial functional imbalance. Moreover, some studies have primarily emphasized mitochondria as targets of injury, without adequately highlighting that mitochondria themselves can also serve as important drivers of secondary brain injury, namely their central role in SBI. Furthermore, although excellent reviews on mitochondrial dysfunction exist in other disease contexts (Liao et al., 2025a), these frameworks cannot be directly and fully applied to ICH due to its unique pathological environment.

**FIGURE 2:**
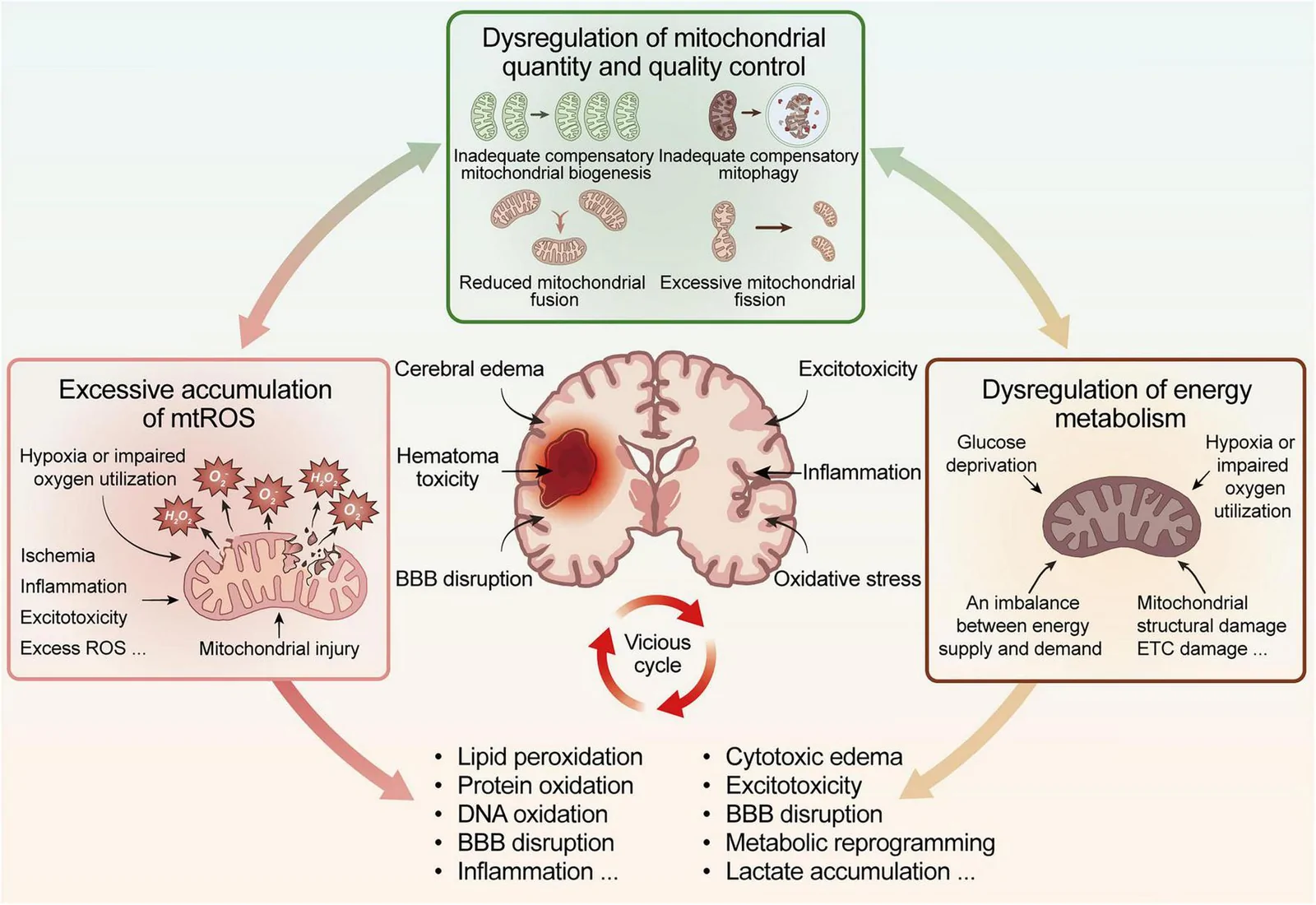
Mitochondrial dysfunction in ICH. Following ICH, mitochondrial dysfunction can be categorized into three major categories: excessive accumulation of mtROS, dysregulation of energy metabolism, and dysregulation of mitochondrial quantity and quality control. These dysfunctions do not occur in isolation; rather, they exhibit extensive crosstalk, thereby amplifying mitochondrial injury and exacerbating SBI.

Therefore, in this review, we propose a mitochondria-centered unifying framework (Figure 2), grounded in the core functions of mitochondria and integrated with the pathological features of ICH. We first categorize post-ICH mitochondrial dysfunction into three major categories: excessive accumulation of mtROS, dysregulation of energy metabolism, and dysregulation of mitochondrial quantity and quality control. Rather than being a mechanical summary of certain mitochondrial processes, this framework focuses on the most central mitochondrial abnormalities, thereby establishing an integrated model that links to the pathological progression of ICH. Finally, based on this framework, we also analyze the mechanistic interactions and intrinsic associations between mitochondrial dysfunction and various forms of cell death after ICH, including apoptosis, necrosis, necroptosis, ferroptosis, and pyroptosis.

This review is primarily informed by ICH-related studies, with Table 1 listing alterations in molecules and pathways that have been directly documented in the ICH literature. Where the underlying mechanisms are relatively conserved and direct ICH evidence is limited, findings from other disease models are used to support the mechanistic discussion, and we explicitly distinguish such extrapolated evidence from ICH-specific data (Table 2).

**TABLE 1:** Molecules and pathways in ICH-related mitochondrial dysfunction.

Molecule / Pathway	Alteration	Cell type(s)	Evidence source	References
O_2_	Decreased (level/utilization)	NA	Human; In vivo	Hemphill et al., 2005; Vespa, 2009; Semyachkina-Glushkovskaya et al., 2016
TNF-α	Increased	Microglia	Human; In vivo	Lan et al., 2017
NO	Increased	NA	Human	Rejdak et al., 2003
MDA	Increased	NA	Human	Yao et al., 2025
4-HNE	Increased	NA	Human; In vivo	Yao et al., 2025
8-OHdG	Increased	NA	Human	Chen et al., 2011; Lorente et al., 2020
MMP	Increased	Microglia	In vivo	Zhang et al., 2022a
AQP4	Decreased	Astrocytes	*In vivo*	Jeon et al., 2021
NLRP3 inflammasome	Activated	Microglia; Astrocytes	*In vivo*; *In vitro*	Luo et al., 2019; Cao et al., 2025; He et al., 2025
AIM2 inflammasome	Activated	Microglia	*In vivo*; *In vitro*	Gu et al., 2024
cGAS–STING pathway	Activated	Astrocytes; Macrophages	Human; *In vivo*; *In vitro*	Andrade et al., 2022; He et al., 2026
NF-κB pathway	Activated	Macrophages	*In vivo*; *In vitro*	Teng et al., 2009; Zhou J. et al., 2018; Herb et al., 2019
MAPK pathway	Activated	NA	*In vivo*	Wu et al., 2022
TLR pathway	Activated	NA	*In vivo*	Shang et al., 2025
Glucose	Decreased	NA	Human	Wang et al., 2008; Tobieson et al., 2019, 2022
Hexokinase	Decreased	Microglia	*In vivo*; *In vitro*	Li Y. et al., 2025
MDH1	Deacetylated	Neurons	*In vivo*; *In vitro*	Wang M. et al., 2022
Cytosolic aspartate aminotransferase	Decreased	NA	*In vivo*	Liu et al., 2018
OGC	Inhibited	NA	*Ex vivo*	Miniero et al., 2022
KGDH	Inhibited	NA	Human	Hansen and Gibson, 2022
PPA2	Decreased	NA	*In vivo*	Liu et al., 2018
Cerebral lactate	Increased	NA	Human; *In vivo*	Mun-Bryce et al., 1993; Zhou Y. et al., 2018; Tobieson et al., 2019, 2022; Liu et al., 2020; Rasulo et al., 2020; Ai et al., 2023; Zhang et al., 2024
VEGF	Increased	Microvascular endothelial cells	*In vivo*	Zhou J. et al., 2018
PGC-1α	Increased	NA	*In vivo*	Zhou Y. et al., 2018; Tang et al., 2025
SIRT1	Increased (total); Decreased (nuclear localization)	NA	*In vivo*	Zhou Y. et al., 2018
Drp1	Translocated	Astrocytes; Neurons	*In vivo*; *In vitro*	Wu et al., 2020a,b
p-Drp1(Ser616)	Increased	Microglia; Astrocytes; Neurons	*In vivo*; *In vitro*	Wu et al., 2020a,b; An et al., 2025
p-Drp1(Ser637)	Decreased	Astrocytes; Neurons	*In vivo*; *In vitro*	Wu et al., 2020a,b; He et al., 2026
TREM-1	Increased	Microglia	Human; *In vivo*; *In vitro*	An et al., 2025
PI3K/Akt pathway	Activated	Microglia	*In vitro*	An et al., 2025
PGAM5	Increased	Astrocytes	Human; *In vivo*; *In vitro*	He et al., 2026
VDAC2	Succinylated	NA	*In vivo*	Liang Y.-J. et al., 2022
Mfn1	Decreased	Neurons	*In vivo*; *In vitro*	Diao et al., 2024
Mfn2	Decreased	Neurons	*In vivo*; *In vitro*	Diao et al., 2024
Opa1	Decreased	Microglia; Neurons	*In vivo*; *In vitro*	Wu et al., 2023c; Diao et al., 2024
PINK1/Parkin pathway	Inhibited	Microglia	*In vivo*	Li et al., 2021
PINK1	Decreased	Microglia	Human; *In vivo*	Li et al., 2021
Parkin	Decreased	Microglia	*In vivo*	Li et al., 2021

This table summarizes representative molecules and signaling pathways reported to be altered after ICH and linked to mitochondrial dysfunction. “Alteration” indicates the direction or status change reported in the cited studies (e.g., increased/decreased expression or activity; activation/inhibition; post-translational modification; subcellular translocation). “Cell type(s)” denotes the major cell population(s) in which the alteration was reported. “NA” indicates that the corresponding cell type(s) were not specified in the cited study/studies. “Evidence source” indicates the experimental context: human (clinical samples/observations), *in vivo* (animal models), *in vitro* (cell culture), and *ex vivo* (isolated tissues/organelles). For entries supported by multiple references, the cell type(s) and evidence source(s) shown represent the collective body of evidence across the cited literature; individual studies may report only a subset of these features.

**TABLE 2:** Evidence context for mitochondrial dysfunction mechanisms.

Mitochondrial dysfunction mechanism	Disease / Model	References
Reduced cerebral blood flow, hematoma mass effect, and elevated intracranial pressure lead to focal cerebral ischemia.	Disease: ICH.	Mendelow et al., 1984; Yang et al., 1994; Xi et al., 2006; Liu T. et al., 2025
During ischemia, forward flux through the TCA cycle is virtually arrested, and SDH operates in reverse to generate large amounts of succinate; upon reperfusion, succinate oxidation drives RET at complex I and leads to massive superoxide generation.	Focal ischemia after ICH. Disease: ICH.	Boveris et al., 1972; Chouchani et al., 2014
	Succinate-driven RET. Disease: ischemia-reperfusion injury; ischemic stroke. Cell model: isolated mitochondria.	
Post-ICH hypoxia or impaired oxygen utilization is reflected by post-ICH hypoxia, HIF-1α responses, reduced oxidative metabolism, or perihematomal oxygen metabolic disturbance.	Disease: ICH; neonatal hemorrhagic stroke.	Zazulia et al., 2001; Jiang et al., 2002; Hemphill et al., 2005; Vespa, 2009; Semyachkina-Glushkovskaya et al., 2016; Wu et al., 2025
During hypoxia or impaired oxygen utilization, COX activity decreases, impeding downstream electron transfer and promoting superoxide anion production.	COX activity decrease. Cell model: rat hepatocyte mitochondria; isolated rat liver mitochondria; bovine heart cytochrome c oxidase.	Turrens et al., 1985; Chandel et al., 1995, 1996; Bleier and Dröse, 2013
	Complex III superoxide generation. Cell model: isolated heart mitochondria.	
Neuroinflammation increases TNF-α and NO; TNF-α reduces complex I activity, and NO inhibits COX activity, thereby increasing mtROS.	TNF-α and NO increase. Disease: ICH.	Cooper, 2002; Rejdak et al., 2003; Tangpong et al., 2008; Prajapati et al., 2015; Lan et al., 2017; Xue and Yong, 2020
	TNF-α-mediated complex I inhibition. Disease: Parkinson disease. Cell model: SH-SY5Y dopaminergic cells; SH-SY5Y-MTRFP cells.	
	NO-mediated COX inhibition. Disease: acute brain injury. Cell model: primary mouse cortical neurons; mouse primary microglia.	
Mitochondrial Ca^2+^ overload triggers sustained mPTP opening, driving mtROS release; ROS themselves can induce sustained mPTP opening and further mtROS release, a process termed ROS-induced ROS release.	Mitochondrial Ca^2+^ overload and mPTP opening. Disease: ICH. Cell model: PC12 cells.	Zorov et al., 2014; Prentice et al., 2015; Song and Dorn, 2015; Bano and Ankarcrona, 2018; Hanafy et al., 2019; Zhang et al., 2022a; Yang et al., 2023
	ROS-induced ROS release. Cell model: cardiac myocytes; isolated mitochondria.	
mtROS initiate lipid peroxidation by oxidizing polyunsaturated fatty acids, triggering a self-amplifying chain reaction.	mtROS promote lipid peroxidation. Disease: ICH.	Porter et al., 1995; Kupsco and Schlenk, 2015; Wan et al., 2019; Liang D. et al., 2022; Yao et al., 2025
	Lipid peroxidation propagates through a chain reaction. Chemical model: unsaturated-lipid autoxidation model.	
mtROS-mediated protein oxidative damage occurs via protein carbonylation, aldehyde-derived cross-linking, disulfide disruption, ER stress, and UPR activation.	Disease: ICH.	Qu et al., 2007; Chen et al., 2010; Mohammed Thangameeran et al., 2020; Cheng et al., 2023; Liu et al., 2024b,c; Yao et al., 2025
mtROS primarily oxidize DNA bases; oxidative damage to mtDNA compromises the ETC, further increasing mtROS accumulation and reducing ATP production.	DNA-base oxidation. Disease: ICH. Cell model: synthetic oligonucleotides and nucleotides; E. coli; COS-7 cells; Saccharomyces cerevisiae.	Kamiya, 2003; Kelly and Scarpulla, 2004; Wallace, 2007; Chen et al., 2011; Anderson et al., 2013; Gorman et al., 2016; Dharmalingam et al., 2020; Lorente et al., 2020
	mtDNA damage and ETC impairment. Disease: mitochondrial disease; ischemic stroke; ICH.	
DNA oxidation activates the cGAS-STING pathway and contributes to neuroinflammation after ICH; DNA oxidation can also induce parthanatos.	cGAS-STING activation after ICH. Disease: ICH. Cell model: astrocytes.	Robinson et al., 2019; Zhang et al., 2022b; He et al., 2026
	DNA oxidation–induced parthanatos. Cell model: macrophages.	
mtROS mediate BBB disruption through MMP activity, AQP4 downregulation, astrocyte dysfunction, and programmed cell death in BBB-associated astrocytes.	Disease: ICH. Cell model: human astrocytes; HBEC-5i human brain endothelial cells in co-culture.	Fang et al., 2020; Jeon et al., 2021; Zhang et al., 2022a
mtROS and released mtDNA promote inflammation by stimulating NLRP3 and AIM2 inflammasomes and activating cGAS-STING, NF-κB, MAPK, and TLR pathways.	NLRP3 and AIM2 inflammasomes activation. Disease: ICH. Cell model: microglia.	García et al., 2005; Teng et al., 2009; Zhang et al., 2010; Herb et al., 2019; Luo et al., 2019; Mandal et al., 2021; Andrade et al., 2022; Wu et al., 2022; Gu et al., 2024; Cao et al., 2025; He et al., 2025, 2026; Shang et al., 2025
	cGAS-STING/NF-κB/TLR signaling. Disease: ICH; traumatic injury. Cell model: astrocytes; macrophages.	
	MAPK signaling. Disease: ICH; hepatocellular carcinoma. Cell model: hepatocellular carcinoma cells.	
mtROS attack guanine bases in RNA and phosphodiester bonds, causing base mispairing, strand breaks, translational errors, impaired protein synthesis, and apoptosis induced by oxidized miRNA.	RNA oxidation. Disease: aging-related oxidative RNA damage. Cell model: human diploid fibroblasts; human lung epithelial cells; mouse brain tissue.	Wang et al., 2015; Alluri et al., 2021; Xu et al., 2021
	Oxidized miRNA-induced apoptosis. Disease: cardiac ischemia/reperfusion injury. Cell model: H9c2 cardiomyocytes.	
Once peroxidized by mtROS, cardiolipin compromises ETC function and inner mitochondrial membrane integrity, facilitates Cyt c release, and externalized cardiolipin can elicit inflammation and contribute to pyroptosis.	CL peroxidation, ETC impairment, IMM disruption, and Cyt c release. Disease: traumatic brain injury. Cell model: primary rat cortical neurons; mechanically stretched neurons; brain mitochondria and brain homogenates.	Rosca et al., 2009; Claypool and Koehler, 2012; Ji et al., 2012; Iyer et al., 2013; Szeto, 2014; Elliott et al., 2018; Miao et al., 2023
	CL externalization and pyroptosis. Model: macrophages; cardiolipin-stained isolated mitochondria; cardiolipin liposome-based membrane systems.	
Oxidation of the endothelial glycocalyx by mtROS increases vascular permeability, promotes tissue inflammation and edema, disrupts microcirculatory hemodynamics, and may precipitate thrombosis.	Disease: subarachnoid hemorrhage; delayed cerebral ischemia; ischemic stroke; Alzheimer disease. Cell model: IDH2-deficient endothelial cells.	Schenck et al., 2021; Balistreri et al., 2024
Alterations in transporters and enzymes impair glycolysis, the malate-aspartate shuttle, the TCA cycle, and OXPHOS, ultimately reducing ATP production.	Disease: ICH. Cell model: microglia; neurons.	Liu et al., 2018; Wang M. et al., 2022; Li Y. et al., 2025
Aberrant upregulation of UCPs can induce mitochondrial uncoupling and reduce ATP production; UCP2 upregulation following ICH may also reduce mtROS production and improve mitochondrial dynamics.	UCP-mediated uncoupling. Disease: ICH; neurodegeneration; brain trauma. Model: brain mitochondria.	Mattiasson et al., 2003; Kim-Han et al., 2006; Augustynek et al., 2014; Wang et al., 2025; Han et al., 2026
	UCP2-mediated changes in mtROS and mitochondrial dynamics. Disease: ICH.	
A metabolic shift from OXPHOS toward glycolysis occurs following ICH.	Disease: ICH. Cell model: microglia.	Wagner et al., 1998; Tobieson et al., 2019, 2022; Rasulo et al., 2020; Xiao et al., 2023; Liu et al., 2024a; Li Y. et al., 2025
Energy failure disrupts ionic gradients maintained by neuronal Na^+^/K^+^-ATPase, promoting depolarization, excitotoxicity, Ca^2 +^ overload, and neuronal death.	Disease: ischemic stroke; ischemic white matter injury.	Li and Stys, 2001; Fricker et al., 2018
In astrocytes, energy failure is manifested by impaired glutamate uptake and disrupted ion/water homeostasis, aggravating excitotoxicity, cytotoxic edema, and BBB disruption.	Disease: ICH.	Gao et al., 2022
In astrocytes, energy failure is manifested by impaired glutamate uptake and disrupted ion/water homeostasis, aggravating excitotoxicity, cytotoxic edema, and BBB disruption.	Disease: amyotrophic lateral sclerosis.	Lee et al., 2012
In microglia and neurovascular cells, energy crisis promotes inflammatory activation and BBB disruption, amplifying SBI.	Disease: ICH.	Lee et al., 2022; Li Y. et al., 2025
Although mitochondrial biogenesis is activated as a compensatory response after ICH, this response is ultimately inadequate due to signaling delays and impaired regulator activity.	Activated mitochondrial biogenesis. Disease: ICH.	Zhou Y. et al., 2018; Wang et al., 2024; Zhang et al., 2025
	Delayed signaling and impaired regulator activity. Disease: surgical brain injury; ICH.	
MAMs provide platforms that facilitate Drp1-dependent fission; VDAC2 alterations after ICH may perturb MAM formation and function.	MAM-facilitated fission. Disease: cerebral ischemia; neurodegenerative diseases.	Friedman et al., 2011; Liang Y.-J. et al., 2022; He et al., 2023; Jiang et al., 2023
	VDAC2 alteration. Disease: ICH.	
Mitochondrial dysfunction participates in intrinsic apoptosis through DNA or RNA damage, mtROS accumulation, UPR activation, Drp1-mediated excessive mitochondrial fission, BAX/BAK-mediated MOMP, Cyt c release, apoptosome assembly, and caspase activation.	UPR-mediated apoptosis. Disease: ICH.	Li et al., 1997; Frank et al., 2001; Wei et al., 2001; Wang et al., 2015; Redza-Dutordoir and Averill-Bates, 2016; Mohammed Thangameeran et al., 2020; Newton et al., 2024
	Other mechanisms of apoptosis. Model: HeLa S-100 fraction; purified Apaf/caspase proteins; primary murine embryonic fibroblasts; mouse hepatocytes.	
Extrinsic apoptosis can be amplified by mitochondrial engagement through caspase-8-mediated BID cleavage and BAX/BAK activation.	Model: mouse liver mitochondria; HeLa S-100 fraction; Xenopus cell-free system; cerebrocortical neurons.	Luo et al., 1998; Budd et al., 2000; Newton et al., 2024
Mitochondrial dysfunction is closely involved in necroptotic signaling; elevated mtROS promotes RIPK1 autophosphorylation, and mtDNA released into the cytosol can be sensed by ZBP1 to activate the RIPK3-MLKL axis.	mtROS-RIPK1 necroptotic signaling. Disease: ICH. Cell model: hemoglobin/hemin-treated neurons	Zhang et al., 2017; Zille et al., 2017; Wang W.-Y. et al., 2022; Lai et al., 2024
	mtDNA-ZBP1-RIPK3/MLKL axis. Disease: developmental sevoflurane neurotoxicity; diquat poisoning.	
Excessive mtROS drive lipid peroxidation and deplete GSH, thereby promoting ferroptosis; inhibiting mitochondrial glutaminolysis can reduce TCA cycle activity and attenuate ferroptosis.	mtROS-driven ferroptosis. Cell model: human fibrosarcoma cells; mouse embryonic fibroblasts; fumarate hydratase-mutant cancer cells.	Gao et al., 2015, 2019
	Glutaminolysis-regulated ferroptosis. Disease: ischemia-reperfusion-induced heart injury. Model: mouse embryonic fibroblasts; isolated mouse hearts.	
Accumulated mtROS and/or released mtDNA can act as danger signals to promote assembly of the NLRP3 or AIM2 inflammasome, thereby triggering pyroptosis.	Disease: ICH. Cell model: microglia.	Luo et al., 2019; Gu et al., 2024

This table summarizes the evidence context for the mitochondrial dysfunction mechanisms discussed in this review. “Disease / Model” indicates the human disease and/or model reported in the studies cited for the corresponding mechanism.

## 2 Literature search strategy

We used a structured multi-stage literature search strategy and searched PubMed, Web of Science, Embase, and Scopus from database inception to June 2026. Search terms were adapted for each database according to its controlled vocabulary and field-tag requirements. Only English-language publications were considered. Two authors (TC and XG) independently screened the titles and abstracts of retrieved records, and potentially relevant articles were then assessed by full-text review. Disagreements were resolved through discussion with a third author (PL).

Stage 1: ICH-specific database search. We performed a structured Boolean search to identify studies directly linking intracerebral hemorrhage (ICH) with mitochondrial dysfunction and related pathological mechanisms. The complete search strategy is shown for PubMed as an example and was adapted for Web of Science, Embase, and Scopus using equivalent controlled vocabulary terms, free-text terms, Boolean operators, and database-specific field tags.

Group A, disease terms:

(“Cerebral Hemorrhage”[Mesh] OR “intracerebral hemorrhage”[tiab] OR “intracerebral haemorrhage”[tiab] OR “cerebral hemorrhage”[tiab] OR “cerebral haemorrhage”[tiab] OR “brain hemorrhage”[tiab] OR “brain haemorrhage”[tiab] OR “intraparenchymal hemorrhage”[tiab] OR “intraparenchymal haemorrhage”[tiab] OR “hemorrhagic stroke”[tiab] OR “haemorrhagic stroke”[tiab] OR ICH[tiab]).

Group B, core mitochondrial dysfunction terms:

(“Mitochondria”[Mesh] OR mitochondria[tiab] OR mitochondrial[tiab] OR mitochondrion[tiab] OR “mitochondrial dysfunction”[tiab] OR “mitochondrial damage”[tiab] OR “mitochondrial injury”[tiab] OR “mitochondrial impairment”[tiab] OR “mitochondrial homeostasis”[tiab] OR “mitochondrial quality control”[tiab] OR “mitochondrial membrane potential”[tiab] OR “mitochondrial permeability transition pore”[tiab] OR “mitochondrial DNA”[tiab] OR “electron transport chain”[tiab] OR “respiratory chain”[tiab] OR “oxidative phosphorylation”[Mesh] OR “oxidative phosphorylation”[tiab]).

Group C, specific categories of mitochondrial dysfunction:

(“reactive oxygen species”[Mesh] OR “reactive oxygen species”[tiab] OR ROS[tiab] OR mtROS[tiab] OR “oxidative stress”[Mesh] OR “oxidative stress”[tiab] OR “energy metabolism”[Mesh] OR “energy metabolism”[tiab] OR ATP[tiab] OR glycolysis[tiab] OR lactate[tiab] OR “metabolic reprogramming”[tiab] OR “mitochondrial uncoupling”[tiab] OR “mitochondrial biogenesis”[tiab] OR “mitochondrial dynamics”[tiab] OR “mitochondrial fission”[tiab] OR “mitochondrial fusion”[tiab] OR mitophagy[tiab] OR “calcium overload”[tiab]).

Group D, pathological consequences and cell death:

(“secondary brain injury”[tiab] OR neuroinflammation[tiab] OR inflammasome[tiab] OR “blood-brain barrier”[Mesh] OR “blood-brain barrier”[tiab] OR BBB[tiab] OR “brain edema”[tiab] OR apoptosis[Mesh] OR apoptosis[tiab] OR necrosis[tiab] OR necroptosis[tiab] OR ferroptosis[tiab] OR pyroptosis[tiab]).

The final PubMed search formula for Stage 1 was: Group A AND Group B AND (Group C OR Group D). This Boolean structure was selected to retrieve studies that simultaneously addressed ICH

Stage 2: Mechanism-oriented supplementary searches. To support the mechanistic framework of this review, targeted supplementary searches were performed for specific topics including fundamental mitochondrial biology, cell death pathways, oxidative stress, energy metabolism, mitochondrial dynamics, mitophagy, and mitochondrial biogenesis. These searches were not restricted to ICH models and included evidence from ischemia, traumatic brain injury, subarachnoid hemorrhage, neurodegenerative diseases, immunological models, cancer biology, and general mitochondrial biology. Non-ICH mechanistic studies were considered eligible only when the pathway was highly conserved and mechanistically well validated, directly relevant to the ICH-related mitochondrial mechanisms discussed in this review, and insufficiently characterized in ICH-specific models. The model or disease context from which these non-ICH mechanistic data were derived is summarized in Table 2, to distinguish foundational mechanistic evidence from direct ICH evidence.

Stage 3: Citation tracking and manual reference screening. The reference lists of included original studies, key review articles, and seminal mechanistic papers were manually examined to identify additional relevant publications. This step was used to capture important classic studies that may have been missed by database searches because of outdated terminology, non-standard indexing, or absence of contemporary keywords.

Inclusion and exclusion criteria. For Stage 1, studies were considered eligible if they met the following criteria: (a) investigated mitochondrial function, mitochondrial injury, or mitochondria-related mechanisms in the context of ICH, including *in vivo* animal models, *ex vivo* tissue preparations, *in vitro* cell models, and human ICH samples or populations, provided that the *in vitro* studies were used specifically to validate molecular mechanisms relevant to ICH pathophysiology; (b) were original research articles or systematic/narrative reviews that provided mechanistic insights; and (c) reported sufficient methodological detail to allow assessment of mitochondrial outcomes. We excluded conference abstracts, case reports, and studies lacking sufficient mechanistic evidence. For Stage 2 non-ICH mechanistic studies, inclusion required that they met the three criteria listed above (highly conserved, directly relevant, insufficiently characterized in ICH). For Stage 3, additional records were incorporated when they provided essential mechanistic, historical, or conceptual support for the mitochondrial framework of this review.

Due to differences in study design, methodology, experimental models, clinical populations, and sources of evidence, the results were synthesized narratively. Accordingly, this review was designed and conducted as a narrative review.

## 3 Excessive accumulation of mtROS

Mitochondria are the predominant source of ROS, generating approximately 90% of cellular ROS (Balaban et al., 2005). The resulting mtROS include superoxide anion (O_2_^–^) and hydrogen peroxide (H_2_O_2_) (Murphy, 2009). Under physiological conditions, electrons are transferred along the mitochondrial electron transport chain (ETC), during which a small fraction leaks from complex I and complex III, generating low levels of mtROS (Saraste, 1999; Turrens, 2003). These physiological mtROS participate in immune regulation and signal transduction (West et al., 2011; Sena and Chandel, 2012).

After ICH, mitochondrial structure and function are directly impaired, leading to excessive mtROS accumulation. These elevated mtROS act as potent oxidants that exacerbate oxidative stress, thereby driving the initiation and progression of SBI (Duan et al., 2016; Shao et al., 2022).

### 3.1 Mechanisms underlying excessive mtROS accumulation

**FIGURE 3:**
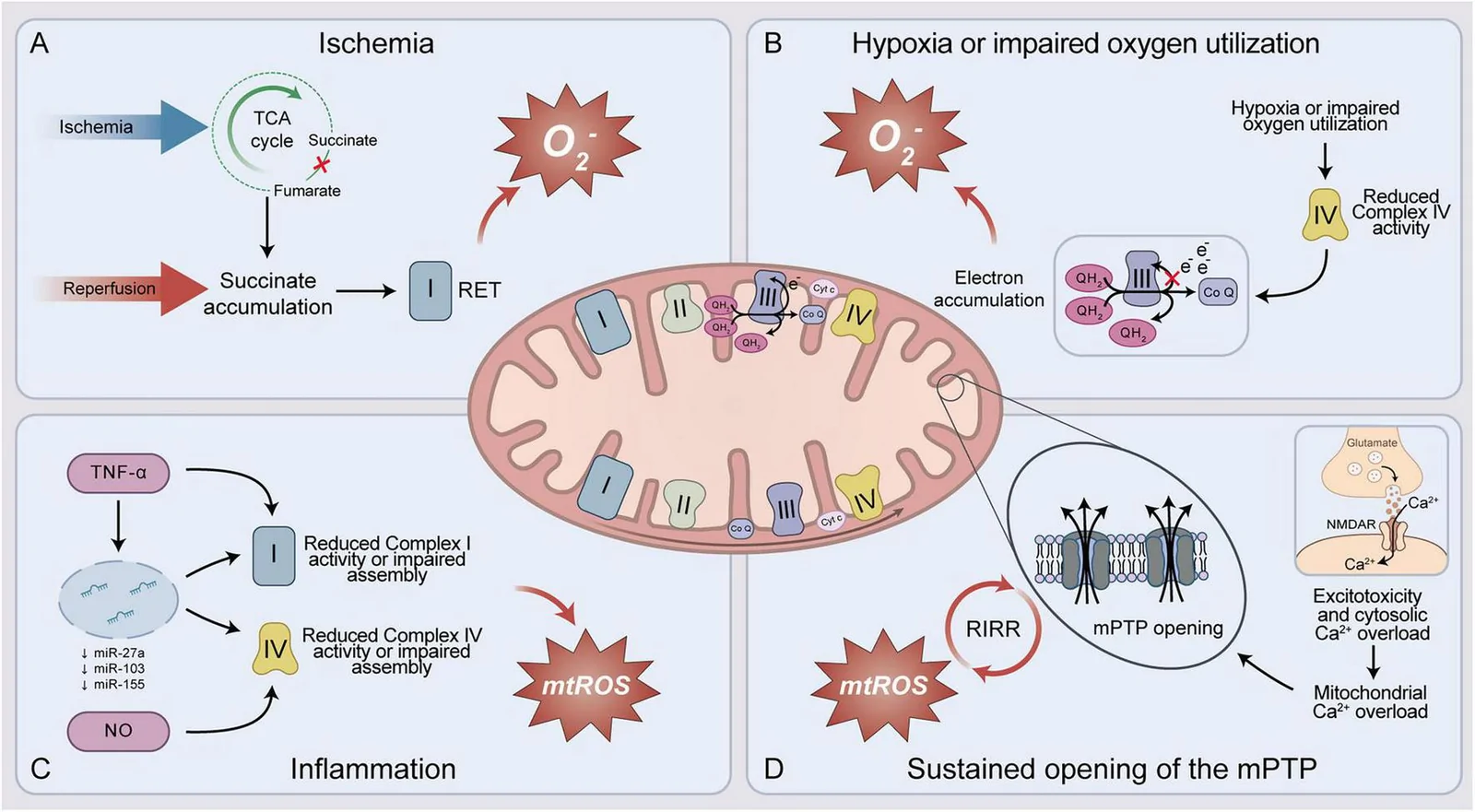
Mechanisms of excessive mtROS accumulation. **(A)** Ischemia inhibits the forward flux of the TCA cycle, leading to succinate accumulation. Upon reperfusion, the TCA cycle resumes forward operation, and the accumulated succinate is rapidly oxidized in mitochondria, driving RET at complex I and resulting in O_2_^–^ production. **(B)** Hypoxia or impaired oxygen utilization reduces COX activity, blocking the entry of electrons generated by the Q cycle into downstream electron transport. The accumulated electrons react with O_2_ to generate O_2_^–^. **(C)** Elevated TNF-α directly reduces complex I activity and downregulates specific miRNAs (e.g., miR-27a, miR-103, and miR-155), thereby impairing the assembly and function of complex I and IV, promoting electron leakage, and increasing mtROS production. NO also inhibits complex IV activity, further increasing mtROS. **(D)** Glutamate excitotoxicity persistently activates postsynaptic NMDARs, leading to cytosolic and mitochondrial Ca^2+^ overload, which promotes sustained opening of the mPTP. Furthermore, ROS can trigger sustained mPTP opening to release further mtROS, a process termed RIRR.

Excessive mtROS accumulation can result from increased production or reduced clearance. This section focuses on mitochondrial mechanisms that increase mtROS production (Figure 3). Although reduced clearance due to impaired antioxidant systems is also important, this process is largely independent of mitochondrial function and is therefore not discussed in detail here.

#### 3.1.1 Ischemia

Under physiological conditions, succinate dehydrogenase (SDH, complex II) catalyzes the oxidation of succinate to fumarate, a key step in the tricarboxylic acid (TCA) cycle. Following ICH, reduced cerebral blood flow, hematoma mass effect, and elevated intracranial pressure lead to focal cerebral ischemia (Table 2; Mendelow et al., 1984; Yang et al., 1994; Xi et al., 2006; Liu T. et al., 2025). During ischemia, forward flux through the TCA cycle is virtually arrested, and SDH operates in reverse, utilizing accumulated ubiquinol as an electron donor to reduce fumarate and generate large amounts of succinate (Figure 3A; Chouchani et al., 2014). This succinate accumulation essentially stores substantial reducing equivalents and high-energy electrons, thereby creating a latent vulnerability to a subsequent mitochondrial oxidative burst (Boveris et al., 1972).

As the disease progresses into the recovery phase, hematoma resolution and decreased intracranial pressure collectively restore local cerebral perfusion. At this stage, the TCA cycle resumes its forward direction, and the accumulated succinate is rapidly oxidized by SDH, driving a massive influx of electrons into the ETC, causing a sharp rise in the proportion and concentration of ubiquinol and a concomitant acute increase in mitochondrial membrane potential. These conditions collectively drive reverse electron transport (RET) at complex I, leading to the massive generation of superoxide anions (Figure 3A; Chouchani et al., 2014).

#### 3.1.2 Hypoxia or impaired oxygen utilization

Whether brain tissue becomes hypoxic after ICH remains a matter of debate. Several studies support the occurrence of post-ICH hypoxia (Hemphill et al., 2005; Semyachkina-Glushkovskaya et al., 2016; Wu et al., 2025), and the upregulation of hypoxia-inducible factors has also been regarded as a marker of hypoxic responses (Jiang et al., 2002; Wu et al., 2025). However, other studies have reached different conclusions. Using positron emission tomography, Zazulia et al. (2001) measured cerebral blood flow (CBF), cerebral metabolic rate of oxygen (CMRO_2_), and oxygen extraction fraction (OEF) in 19 patients with ICH 5–22 h after onset. They found that the reduction in CMRO_2_ exceeded that in CBF, leading to a decreased OEF, a pattern not consistent with classical tissue hypoxia. Vespa (2009) similarly suggested that reduced OEF argues against severe acute hypoxia. Nevertheless, these findings should be interpreted cautiously. The study by Zazulia et al. had a small sample size, included predominantly patients without marked mass effect, and assessed only an early time window, which may limit its generalizability across different severities and stages of ICH. Vespa (2009) argued against acute severe hypoxia but noted that inflammation, epileptiform discharges, and spreading depolarizations may exacerbate local oxygen supply–demand mismatch, creating focal or transient hypoxic microenvironments. Notably, both studies consistently demonstrated reduced oxidative metabolism in perihematomal tissue after ICH. This finding suggests that impaired oxygen utilization can occur even when oxygen delivery remains adequate (Table 1).

During hypoxia or impaired oxygen utilization, cytochrome c oxidase (COX; complex IV) exhibits an increase in the Michaelis constant for its substrates—cytochrome c (Cyt c) and O_2_—and a decrease in the maximum reaction velocity. This indicates reduced substrate affinity and diminished catalytic capacity of COX under low oxygen conditions, likely due to hypoxia-induced conformational changes that impair its activity (Chandel et al., 1995, 1996). COX is located at the terminus of the ETC. A decrease in its activity impedes the downstream transfer of electrons generated by the upstream Q cycle, and the accumulated electrons react with O_2_ to produce superoxide anions (Figure 3B; Turrens et al., 1985; Bleier and Dröse, 2013).

#### 3.1.3 Inflammation

Following ICH, the toxicity of hematoma components, microglial M1 polarization, and infiltration of peripheral immune cells can all trigger neuroinflammation, which in turn increases tumor necrosis factor-α (TNF-α) levels (Lan et al., 2017; Xue and Yong, 2020). Elevated TNF-α can directly reduce complex I activity (Prajapati et al., 2015). Because complex I is the primary entry point for electrons into the ETC, its inhibition promotes electron leak to O_2_ and increases superoxide production (Prajapati et al., 2015). TNF-α can also impair mitochondrial function by downregulating specific miRNAs, including miR-27a, miR-103, and miR-155, that normally sustain the expression of nuclear-encoded subunits of complex I and COX (Prajapati et al., 2015); their loss ultimately disrupts complex assembly and increases mtROS production. Nitric oxide (NO), another inflammatory mediator markedly elevated after ICH (Rejdak et al., 2003), competes with oxygen for binding to the active site of COX (the heme a3–CuB binuclear center), thereby inhibiting COX activity (Cooper, 2002; Tangpong et al., 2008). This results in upstream electron accumulation and a consequent increase in mtROS (Figure 3C).

#### 3.1.4 Sustained opening of the mPTP

Mitochondria are double-membrane organelles that confine intense redox reactions within the matrix and limit excessive leakage of the respiratory by-product mtROS into the cytosol (Song and Dorn, 2015). The mPTP resides in the inner mitochondrial membrane (IMM), and its sustained opening promotes mtROS release and drives tissue injury (Figure 3D; Yang et al., 2023).

Mitochondrial Ca^2+^ overload after ICH triggers sustained mPTP opening, driving mtROS release (Yang et al., 2023). The primary mechanism involves glutamate excitotoxicity: excess glutamate released after ICH persistently activates postsynaptic N-methyl-D-aspartate receptors (NMDARs), drives substantial Ca^2+^ influx, and promotes mitochondrial Ca^2+^ uptake, culminating in mitochondrial Ca^2+^ overload (Prentice et al., 2015; Bano and Ankarcrona, 2018; Zhang et al., 2022a). Furthermore, ROS themselves can induce sustained mPTP opening and further mtROS release, a process termed ROS-induced ROS release (RIRR) (Figure 3D; Zorov et al., 2014). Following ICH, the initiating ROS sources that trigger RIRR are diverse, encompassing mtROS described above (arising from ischemia, hypoxia, impaired oxygen utilization, inflammation, or Ca^2+^ overload) as well as non-mitochondrial ROS, such as hydroxyl radicals generated by the Fenton reaction (Hanafy et al., 2019; Tang D. et al., 2021).

### 3.2 Pathological consequences mediated by mtROS

**FIGURE 4:**
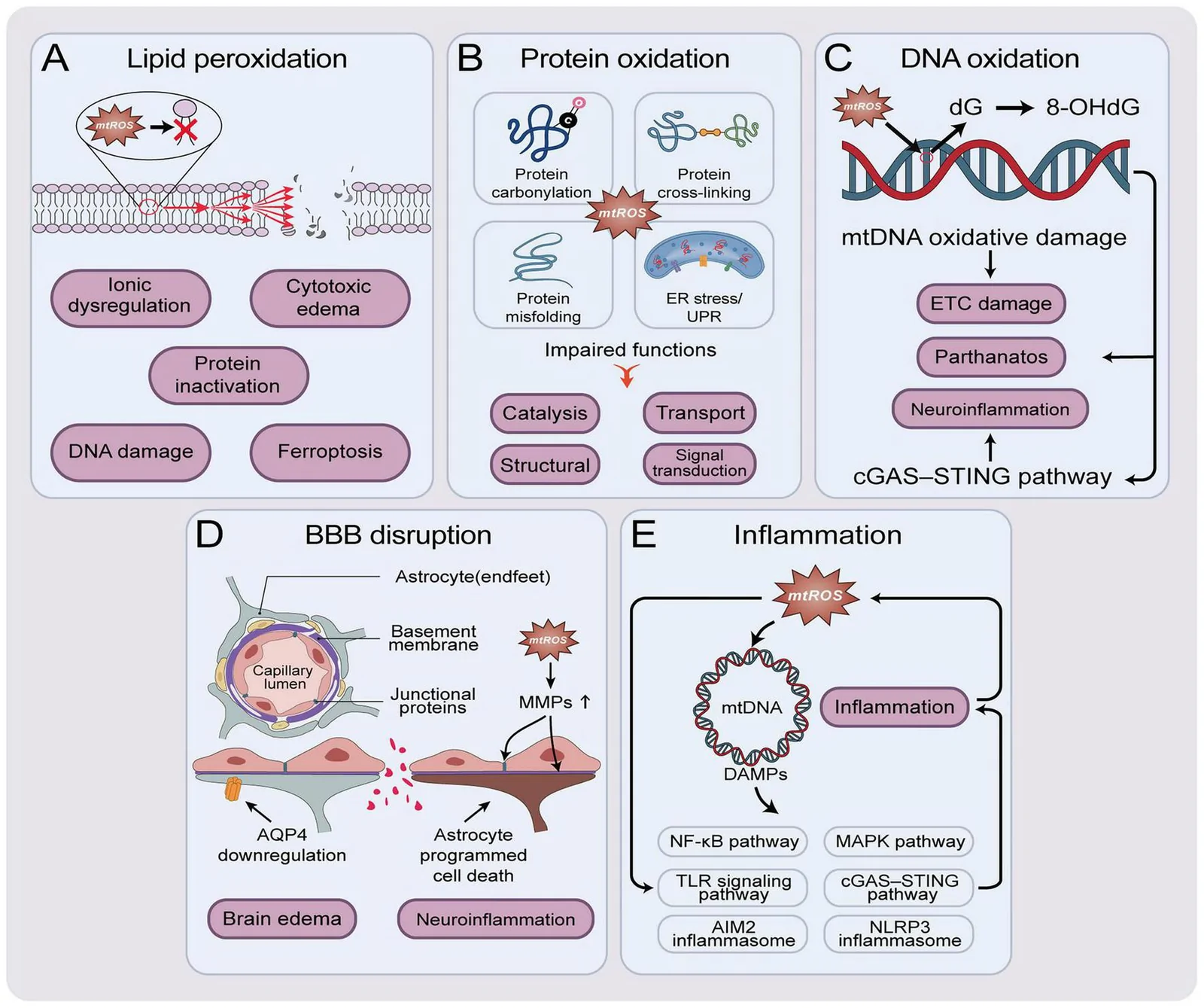
Pathological consequences mediated by mtROS. **(A)** Excessive mtROS trigger lipid peroxidation, compromising cell membranes to cause ionic dysregulation and cytotoxic edema. This process also generates cytotoxic aldehydes that lead to protein inactivation and DNA damage, and impairs antioxidant systems to drive ferroptosis. **(B)** mtROS induce protein carbonylation, protein cross-linking, and protein misfolding, which can subsequently trigger ER stress and the UPR. These oxidative damages impair diverse biological functions of proteins. **(C)** mtROS oxidize DNA bases, producing lesions such as 8-OHdG (via the oxidation of dG). DNA oxidation can activate the cGAS–STING pathway to promote neuroinflammation and induce parthanatos. Additionally, oxidative damage to mtDNA compromises the ETC. **(D)** mtROS upregulate MMPs, which degrade the basement membrane and junctional proteins. They also cause AQP4 downregulation and programmed cell death in astrocytes. These events disrupt the BBB, exacerbating brain edema and neuroinflammation. **(E)** mtROS and released mtDNA act as DAMPs to activate inflammatory responses. They stimulate the NLRP3 and AIM2 inflammasomes and activate multiple pro-inflammatory pathways, including NF-κB, MAPK, TLR, and cGAS–STING. Ultimately, inflammation and mtROS form a reciprocal, self-amplifying vicious cycle.

mtROS-mediated injury is pervasive, encompassing oxidative damage to lipids, proteins, and DNA, as well as the promotion of inflammation and BBB disruption (Figure 4).

#### 3.2.1 Lipid peroxidation

mtROS initiate lipid peroxidation by oxidizing polyunsaturated fatty acids, triggering a self-amplifying chain reaction that propagates unless terminated by antioxidants or radical recombination (Porter et al., 1995; Kupsco and Schlenk, 2015). In the context of ICH, lipid peroxidation exerts multiple deleterious effects (Figure 4A). First, the plasma and organelle membranes of brain cells (including neurons, endothelial cells, and glial cells) are enriched in polyunsaturated fatty acids. Their oxidation compromises membrane integrity, fluidity, and permeability, leading to ionic dysregulation and cytotoxic edema (Wan et al., 2019). Second, lipid peroxides are unstable and can decompose into cytotoxic aldehydes, such as 4-hydroxynonenal (4-HNE) and malondialdehyde (MDA), which form cross-links with proteins or DNA, resulting in protein inactivation or DNA damage (Yao et al., 2025). Finally, lipid peroxidation impairs the glutathione peroxidase-mediated antioxidant system, thereby driving ferroptosis (Liang D. et al., 2022).

#### 3.2.2 Protein oxidation

Following ICH, mtROS-mediated protein oxidative damage occurs via three major mechanisms (Figure 4B). First, mtROS directly oxidize amino acid side chains, leading to protein carbonylation (Qu et al., 2007; Xu and Chance, 2007; Chen et al., 2010). Second, cytotoxic aldehydes (e.g., 4-HNE and MDA) derived from mtROS-driven lipid peroxidation form cross-links with proteins (Posta et al., 2020; Cheng et al., 2023; Yao et al., 2025). Third, mtROS impair proper protein folding by disrupting disulfide bonds or inhibiting Ca^2+^-ATPases, thereby leading to the inactivation of enzymes and critical signaling molecules (Kupsco and Schlenk, 2015). If such oxidative stress causes the accumulation of misfolded proteins in the ER, it can further induce ER stress and activate the unfolded protein response (UPR) (Kupsco and Schlenk, 2015; Mohammed Thangameeran et al., 2020; Liu et al., 2024b,c).

Collectively, protein oxidation alters the native structure, impairing diverse biological functions—including catalysis, transport, structural support, and signal transduction—and ultimately contributing to widespread cellular dysfunction after ICH (Figure 4B).

#### 3.2.3 DNA oxidation

mtROS primarily oxidize DNA bases, thereby altering base properties and causing mispairing during DNA replication (such as G/T transversions) (Kamiya, 2003). In ICH, the most common site of mtROS-mediated DNA oxidation is the C8 position of guanine (Figure 4C), producing the canonical oxidative lesion 8-hydroxy-2’-deoxyguanosine (8-OHdG) (Chen et al., 2011; Lorente et al., 2020). Studies have reported elevated 8-OHdG levels after ICH that correlate with higher mortality, providing clinical support for the relevance of this mechanism (Lorente et al., 2020). Beyond serving as a marker of oxidative damage, DNA oxidation also promotes SBI. Recent studies have demonstrated that DNA oxidation activates the cyclic GMP–AMP synthase–stimulator of interferon genes (cGAS–STING) pathway and contributes to neuroinflammation after ICH (He et al., 2026). DNA oxidation can also induce parthanatos (Robinson et al., 2019), which may contribute to ICH-related injury (Zhang et al., 2022b).

Notably, eukaryotic cells harbor both nuclear DNA and mitochondrial DNA (mtDNA). mtDNA is particularly vulnerable to damage accumulation due to the absence of protective histones and limited repair capacity (Gorman et al., 2016). In ICH-related studies, Anderson et al. (2013) identified mtDNA mutations, particularly in genes encoding ETC complex IV. Dharmalingam et al. (2020) observed that oxidative stress induces double-strand breaks in both nuclear DNA and mtDNA. mtDNA primarily encodes ETC-related proteins (Kelly and Scarpulla, 2004), and mtDNA damage compromises the ETC (Figure 4C), further increasing mtROS accumulation and reducing ATP production (Wallace, 2007).

#### 3.2.4 BBB disruption

The BBB is a key component of the neurovascular unit and is formed by brain microvascular endothelial cells, pericytes, and astrocytic endfeet, supported by a basement membrane and sealed by tight junction proteins (Figure 4D; Wu et al., 2023a). Functionally, the BBB selectively regulates substance exchange between the peripheral circulation and the brain parenchyma, restricting the entry of harmful substances to maintain central nervous system (CNS) homeostasis (Wu et al., 2023a). Disruption of the BBB facilitates the infiltration of peripheral cells and inflammatory cytokines into the brain, thereby precipitating brain edema and neuroinflammation (Zhao et al., 2006).

In ICH, mtROS mediate BBB disruption through two principal mechanisms. First, mtROS upregulate matrix metalloproteinase (MMP) activity (Zhang et al., 2022a), which degrades the basement membrane and tight junction proteins and thereby compromises BBB structural integrity (Rosenberg, 2009). Second, mtROS directly injure BBB constituents by downregulating aquaporin-4 (AQP4) to impair astrocyte function or by inducing programmed cell death in BBB-associated astrocytes, thereby contributing to BBB disruption (Figure 4D; Fang et al., 2020; Jeon et al., 2021).

#### 3.2.5 Inflammation

Following ICH, mtROS not only promote inflammation via BBB disruption but can also directly activate inflammatory responses by acting as danger signals (Cao et al., 2025). In addition, mtROS can induce mPTP opening, releasing mtDNA into the cytosol (García et al., 2005). As a damage-associated molecular pattern (DAMP), cytosolic mtDNA can further activate inflammatory responses. Specifically, accumulated mtROS and released mtDNA promote inflammation by stimulating the assembly of the NLR family pyrin domain containing 3 (NLRP3) inflammasome and the absent in melanoma 2 (AIM2) inflammasome (Cao et al., 2025; He et al., 2025; Luo et al., 2019; Gu et al., 2024). They also activate multiple pro-inflammatory signaling pathways, including cGAS–STING (Andrade et al., 2022; He et al., 2026), nuclear factor kappa-light-chain-enhancer of activated B cells (NF-κB) (Teng et al., 2009; Herb et al., 2019), mitogen-activated protein kinase (MAPK) (Wu et al., 2022; Mandal et al., 2021), and Toll-like receptor (TLR) pathways (Zhang et al., 2010; Shang et al., 2025). These pathways exhibit extensive crosstalk; for instance, NF-κB activation upregulates NLRP3, amplifying the inflammatory response (Figure 4E; Cao et al., 2025). Collectively, inflammation can drive mtROS accumulation, whereas mtROS can in turn activate inflammation through multiple routes. This reciprocal, self-amplifying loop is a central contributor to SBI after ICH.

It should be emphasized that this review focuses specifically on CNS inflammation after ICH. The inflammatory cascades discussed herein are primarily localized to the CNS compartment, including the brain parenchyma and neurovascular unit, and should be distinguished from inflammatory processes originating from the peripheral nervous system or systemic inflammatory conditions.

#### 3.2.6 Other potential injury pathways and future research directions

##### 3.2.6.1 RNA oxidation

Similar to DNA oxidation, mtROS attack guanine bases in RNA and oxidize the C8 position to form 8-oxoguanosine, which can cause base mispairing (Xu et al., 2021). mtROS can also attack RNA phosphodiester bonds, causing strand breaks and generating RNA fragments (Alluri et al., 2021). Oxidation of mRNA, tRNA, and rRNA can cause translational errors and impair protein synthesis, and oxidation of miRNA has been shown to induce apoptosis (Wang et al., 2015).

Currently, direct evidence for oxidative RNA damage in ICH remains limited, and our understanding is largely informed by studies in ischemic stroke, traumatic brain injury, and subarachnoid hemorrhage. Future ICH studies should directly quantify RNA oxidation in perihematomal tissue by liquid chromatography–tandem mass spectrometry (LC-MS/MS) to analyze oxidized ribonucleosides, complemented by enrichment- and sequencing-based approaches to map 8-oxoguanosine across the transcriptome. Coupling these measurements with ribosome profiling, proteomics, and cell-type-resolved analyses may clarify whether oxidative RNA damage drives translational dysfunction and cell injury after ICH.

##### 3.2.6.2 Cardiolipin peroxidation

Cardiolipin (CL), a unique diphosphatidylglycerol predominantly localized to the IMM, is increasingly recognized as an injury-associated lipid and a potential therapeutic target (Claypool and Koehler, 2012; Szeto, 2014). Under physiological conditions, CL stabilizes ETC complexes and helps maintain the folded architecture of mitochondrial cristae (Szeto, 2014). Once peroxidized by mtROS, CL compromises ETC function and IMM structural integrity, thereby reducing mitochondrial ATP production (Claypool and Koehler, 2012). Furthermore, CL serves as an anchoring site for cytochrome c (Cyt c) on the IMM (Rosca et al., 2009). Under oxidative stress, peroxidized CL facilitates Cyt c release into the cytosol, where Cyt c promotes apoptosome assembly via apoptotic protease-activating factor 1 (Apaf-1) and initiates the apoptotic cascade (Ji et al., 2012). Studies indicate that ROS can induce the externalization of CL to the outer mitochondrial membrane (OMM) (Elliott et al., 2018), which can elicit inflammation and contribute to pyroptosis (Iyer et al., 2013; Miao et al., 2023). In animal models of traumatic brain injury, inhibiting CL peroxidation markedly reduces neuronal apoptosis and lesion volume (Ji J. et al., 2015). A separate study in cerebellar granule neurons showed that anthocyanins alleviate mitochondria-derived oxidative stress, ameliorate CL damage, and exert neuroprotective effects (Kelsey et al., 2011).

Although these findings highlight CL as an important target in neurological disorders, direct evidence in ICH remains limited. Future studies should use targeted LC–MS/MS-based lipidomics to directly quantify oxidized CL species in perihematomal tissue after ICH and assess enzymes or molecules involved in CL remodeling, such as tafazzin and acyl-CoA:lysocardiolipin acyltransferase 1 (Xu et al., 2003; Cao et al., 2004). Compounds with putative cardiolipin-protective effects, such as Szeto-Schiller peptide 31 and anthocyanins, could also be tested in ICH models (Kelsey et al., 2011; Szeto, 2014).

##### 3.2.6.3 Endothelial glycocalyx injury

The glycocalyx is a polysaccharide–protein meshwork that coats the outer surface of the cell membrane (Choi et al., 2022), and the glycocalyx covering vascular endothelial cells is termed the endothelial glycocalyx (eGCX). Functionally, the eGCX is indispensable for vascular homeostasis, acting as a physical barrier that regulates capillary permeability, modulates vascular tone, and exerts anticoagulant effects (Schenck et al., 2021; Villalba et al., 2021). Oxidation of the eGCX by mtROS increases vascular permeability, promotes tissue inflammation and edema, disrupts microcirculatory hemodynamics, and may even precipitate thrombosis (Villalba et al., 2021).

In subarachnoid hemorrhage, oxidative injury to the eGCX has been shown to disrupt the BBB and trigger delayed cerebral ischemia (Schenck et al., 2021). Similarly, eGCX oxidative damage has been implicated in neuroinflammation and vascular injury in Alzheimer’s disease and ischemic stroke (Balistreri et al., 2024). Given the shared pathophysiological features of oxidative stress and vascular dysfunction across these neurological disorders, mtROS-driven eGCX oxidation likely also occurs in ICH, where it may mediate BBB disruption and neuroinflammation and may even be involved in the transition from hemorrhage to ischemia.

Future studies should directly assess eGCX integrity in perihematomal microvessels after ICH using transmission electron microscopy in conjunction with rapid freezing/freeze-substitution and Alcian blue or other cationic staining methods to evaluate glycocalyx thickness and continuity at the ultrastructural level (Reitsma et al., 2007). Concurrently, levels of shed glycocalyx components—including syndecan-1, heparan sulfate, and hyaluronan—could be quantified in perihematomal tissue homogenates or cerebrospinal fluid as biochemical indicators of eGCX degradation (Becker et al., 2015). Furthermore, *in situ* heparinase digestion prior to cationic staining or immunolabeling may help confirm the specificity of heparan sulfate-related signals, thereby distinguishing them from other sulfated glycosaminoglycans and ruling out non-specific electrostatic interactions (Chappell et al., 2008).

## 4 Dysregulation of energy metabolism

As one of the most energy-demanding organs in the body, the brain relies heavily on mitochondria for continuous and efficient energy production (Park et al., 2021). Cellular energy production proceeds through three major stages. First, glycolysis in the cytosol breaks down glucose into pyruvate, generating small amounts of ATP and nicotinamide adenine dinucleotide (NADH) (Chandel, 2021). Second, pyruvate undergoes oxidative decarboxylation to form acetyl coenzyme A, which enters the TCA cycle and is fully oxidized to CO_2_, generating substantial amounts of NADH and flavin adenine dinucleotide (FADH_2_) (Martínez-Reyes and Chandel, 2020). Third, electrons from NADH and FADH_2_ fuel oxidative phosphorylation (OXPHOS) to produce the bulk of ATP (Mitchell, 1961; Saraste, 1999). In brain cells, particularly neurons, the malate–aspartate shuttle serves as the primary auxiliary system. It transfers the reducing equivalents of cytosolic NADH to malate, which enters the mitochondrial matrix and is reoxidized, thereby delivering reducing power to mitochondria (McKenna et al., 2006; Satrústegui and Bak, 2015). Following ICH, this tightly regulated energy metabolism system becomes severely disrupted, which contributes to the progression of SBI.

### 4.1 Mechanisms underlying dysregulation of energy metabolism

#### 4.1.1 Glucose insufficiency and hypoxia or impaired oxygen utilization

The brain relies almost exclusively on glucose as its metabolic fuel (Dienel, 2019). Microdialysis studies after ICH have shown that glucose concentrations in perihematomal regions are lower than in normal brain tissue, indicating glucose insufficiency and a consequent reduction in energy production (Wang et al., 2008; Tobieson et al., 2019, 2022). Notably, although glucose levels decrease, they remain above the critical threshold for energy failure (greater than 0.2 mmol/L and often exceeding 0.8 mmol/L), suggesting that glucose insufficiency may only modestly impair energy production after ICH (Tobieson et al., 2019, 2022).

Energy production also depends on adequate oxygen availability. Mitochondria consume the majority of cerebral oxygen, with approximately 80% used directly for ATP production (Rolfe and Brown, 1997). During hypoxia or impaired oxygen utilization, OXPHOS is inevitably compromised, leading to reduced ATP production (Wilson et al., 1988). Furthermore, should the energy yield from OXPHOS fall below metabolic demand, this shortfall can trigger metabolic reprogramming.

#### 4.1.2 Alterations in transporters and enzymes

During cellular respiration, glycolysis, the malate–aspartate shuttle, the TCA cycle, and OXPHOS depend on enzymatic catalysis and transporter-mediated substrate translocation. Following ICH, alterations in transporters and enzymes contribute to dysregulation of energy metabolism. Specifically, downregulation of hexokinase compromises glycolysis in microglia (Li Y. et al., 2025); deacetylation of malate dehydrogenase 1 (MDH1) or downregulation of cytosolic aspartate aminotransferase impair malate–aspartate shuttle function (Wang M. et al., 2022; Liu et al., 2018); reduced activity of the mitochondrial 2-oxoglutarate carrier (OGC) concurrently limits the shuttle system and TCA cycle substrate supply (Miniero et al., 2022); inhibited activity of α-ketoglutarate dehydrogenase (KGDH) reduces TCA cycle efficiency (Hansen and Gibson, 2022); and downregulation of inorganic pyrophosphatase 2 (PPA2) reduces OXPHOS substrates (Liu et al., 2018). Collectively, these alterations in transporters and enzymes impair multiple stages of energy metabolism, ultimately reducing ATP production.

#### 4.1.3 Damage to mitochondrial structure and the ETC

Within mitochondria, energy production relies on the TCA cycle and OXPHOS. The TCA cycle operates in the mitochondrial matrix and supplies reducing equivalents for OXPHOS (Martínez-Reyes and Chandel, 2020). OXPHOS proceeds across mitochondrial compartments: first, the ETC pumps protons from the matrix into the intermembrane space during electron transfer, thereby establishing a transmembrane proton gradient; subsequently, protons flow back into the matrix down this gradient, driving ATP synthase to generate ATP (Mitchell, 1961; Abrahams et al., 1994). Efficient ATP generation therefore depends on intact mitochondrial architecture and ETC integrity.

As discussed in section “3 Excessive accumulation of mtROS,” multiple post-ICH pathological factors can directly reduce ETC complex activity. In addition, excessive mtROS accumulation induces oxidative damage to lipids, DNA, and proteins and promotes mPTP opening, causing structural mitochondrial injury and ultimately resulting in insufficient ATP production. Moreover, evidence indicates that hematoma-derived toxicity, oxidative stress, and dysregulated mitochondrial dynamics disrupt cristae architecture (Lakovic et al., 2014; Huang et al., 2024). Cristae concentrate ETC complexes and sustain a locally high proton-motive force (Cogliati et al., 2013; Adams et al., 2025). Once cristae architecture is compromised, electron transfer efficiency declines markedly, thereby diminishing OXPHOS efficiency and ATP production.

#### 4.1.4 Mitochondrial uncoupling

In mitochondria, dissipation of the proton gradient without concomitant ATP synthesis is termed uncoupling, a process mediated by uncoupling proteins (UCPs) (Krauss et al., 2005). Under physiological conditions, changes in UCP expression help regulate body temperature in response to cold exposure (Cannon and Nedergaard, 2004). In pathological settings, aberrant upregulation of UCPs can induce mitochondrial uncoupling, thereby reducing ATP production (Krauss et al., 2005). Three UCP isoforms have been identified in the brain: UCP2, UCP4, and UCP5 (Mattiasson et al., 2003). Current research on neurological diseases predominantly focuses on UCP2 (Mattiasson et al., 2003).

Studies indicate that UCP2 is upregulated following ICH, leading to mitochondrial uncoupling (Augustynek et al., 2014; Wang et al., 2025). By assessing mitochondrial respiratory states, Kim-Han et al. (2006) likewise confirmed the presence of uncoupling. However, no studies to date have shown that post-ICH UCP2 upregulation worsens SBI; instead, evidence indicates that increased UCP2 expression alleviates SBI (Wang et al., 2025; Han et al., 2026). This neuroprotective effect is attributed to the ability of UCP2 to reduce mtROS production and improve mitochondrial dynamics (Mattiasson et al., 2003; Wang et al., 2025; Han et al., 2026). This seemingly paradoxical finding is likely explained by the benefits of UCP2 upregulation—particularly reduced oxidative stress—outweighing the cost of diminished ATP synthesis. Nonetheless, this does not negate the adverse effects of uncoupling on energy metabolism and ATP production.

#### 4.1.5 Metabolic reprogramming

Following ICH, multiple factors limit mitochondrial ATP production. Meanwhile, excitotoxicity (Wagner et al., 1998), ionic homeostasis disruption (Wagner et al., 1998), inflammatory activation, oxidative stress management, and cellular repair all increase energy demand, and this energy supply–demand imbalance can drive metabolic reprogramming. Microdialysis studies show that post-ICH brain tissue exhibits elevated lactate and reduced pyruvate, with a corresponding increase in the lactate-to-pyruvate ratio (Tobieson et al., 2019, 2022; Rasulo et al., 2020), indicating a shift in metabolic flux from OXPHOS toward glycolysis. Metabolomics and deuterium metabolic imaging studies further support this shift (Xiao et al., 2023; Liu et al., 2024a). Although evidence suggests that glycolysis is impaired in microglia after ICH, this likely reflects a cell-type-specific metabolic change rather than a tissue-wide phenomenon. At the whole-brain tissue level, glycolytic activity remains increased (Li Y. et al., 2025).

Compared with OXPHOS, glycolysis has a markedly lower ATP yield (2 ATP vs. 30–32 ATP per glucose molecule) (Warburg, 1956; Yetkin-Arik et al., 2019). Given that OXPHOS provides over 90% of cellular ATP (Rolfe and Brown, 1997), even a compensatory shift toward glycolysis would be insufficient to sustain the brain’s energetic demands. However, increased glycolysis may not be entirely detrimental. First, glycolysis produces ATP far faster than OXPHOS (Pfeiffer et al., 2001), a rate advantage that may better accommodate the sudden local surge in energy demand after ICH. Second, high glycolytic flux can promote the accumulation of glucose-6-phosphate, thereby shunting carbon flux into the pentose phosphate pathway and markedly increasing the production of reduced nicotinamide adenine dinucleotide phosphate (NADPH), which is essential for maintaining cellular redox homeostasis (Vander Heiden et al., 2009; Anastasiou et al., 2011). Third, according to the astrocyte–neuron lactate shuttle model, lactate produced by highly glycolytic astrocytes can serve as an energy substrate for neurons (Bélanger et al., 2011). Finally, when OXPHOS is compromised, a metabolic shift toward glycolysis may represent an adaptive strategy by brain cells.

Taken together, a metabolic shift from OXPHOS toward glycolysis occurs following ICH. The mechanisms by which this metabolic reprogramming contributes to energy dysregulation should be understood not solely in terms of insufficient ATP generation, but also with full consideration of potential adaptive responses. However, when the adverse effects—such as reduced ATP yield and lactate-induced toxicity—are weighed against the potential benefits outlined above, the net impact of this metabolic reprogramming remains unclear. Further investigation is warranted at both the mechanistic and clinical levels.

### 4.2 Pathological consequences of dysregulation of energy metabolism

#### 4.2.1 Energy crisis

**FIGURE 5:**
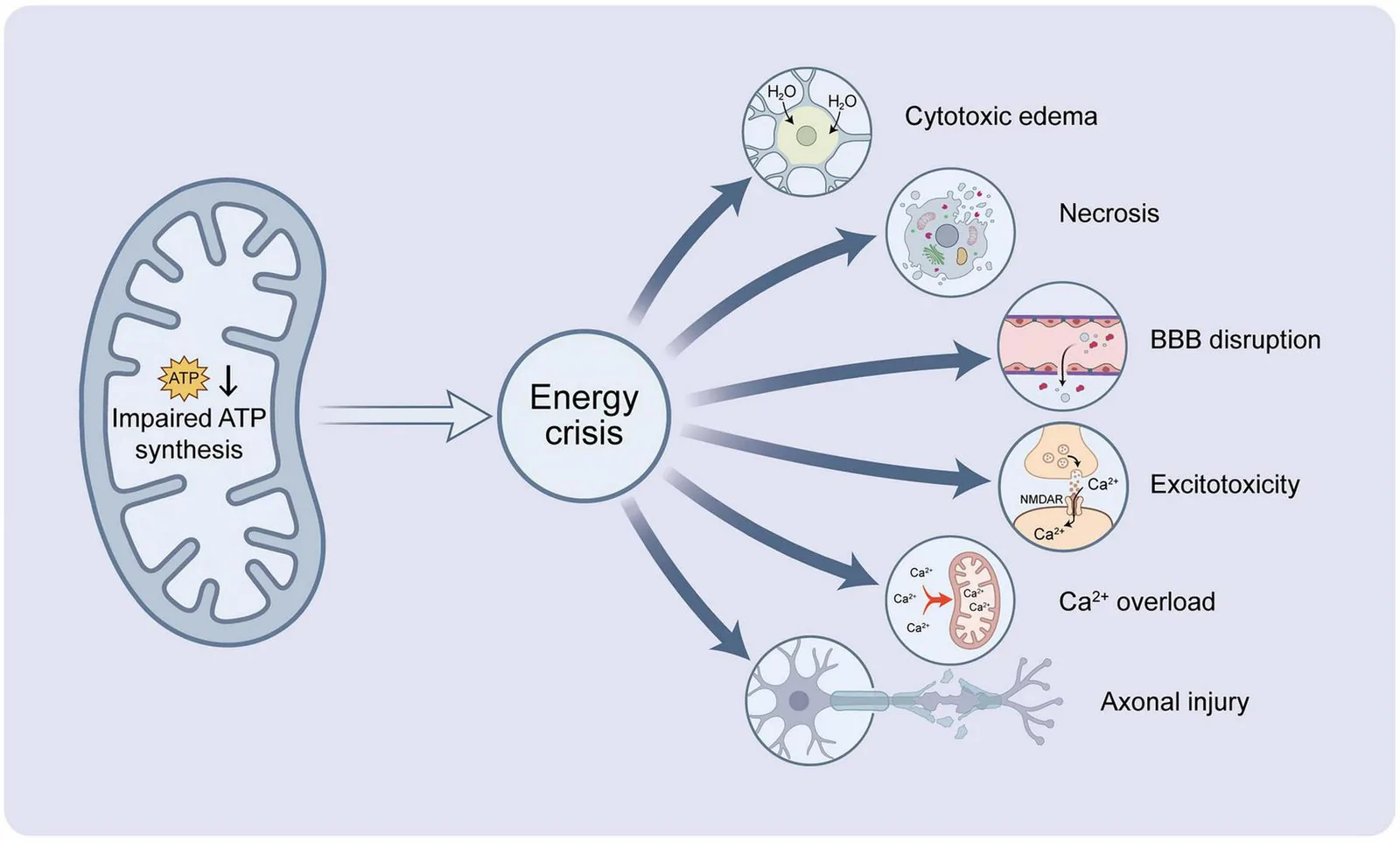
Pathological consequences of the energy crisis. Impaired mitochondrial ATP synthesis leads to a severe energy crisis, which results in cytotoxic edema, necrosis, BBB disruption, excitotoxicity, Ca2+ overload, and axonal injury.

In the brain, different cell types respond differently to an energy crisis, leading to distinct pathological consequences (Figure 5). Neurons are highly vulnerable because of their high ATP turnover, the substantial energy demand required to maintain ionic gradients, and their dependence on glutamate- and Ca^2+^-mediated excitatory signaling (Fricker et al., 2018; Yu et al., 2018). Energy failure can disrupt the ionic gradients maintained by neuronal Na^+^/K^+^-ATPase, promoting membrane depolarization, glutamate excitotoxicity, Ca^2+^ overload, and ultimately neuronal death, including necrosis (Li and Stys, 2001; Fricker et al., 2018). In astrocytes, energy failure mainly manifests as impaired glutamate uptake and disrupted ion and water homeostasis, which aggravate excitotoxicity, cytotoxic edema, and BBB disruption (Gao et al., 2022). Oligodendrocytes are responsible for myelin maintenance and axonal metabolic support, and ATP deficiency can therefore lead to axonal injury (Lee et al., 2012). In microglia and neurovascular cells, an energy crisis promotes inflammatory activation and BBB disruption, further amplifying SBI (Lee et al., 2022; Li Y. et al., 2025).

#### 4.2.2 Lactate accumulation

In most cases, lactate accumulation after ICH is associated with a poor prognosis, as it can lead to acidosis and reflects severe tissue hypoxia (Lehmann et al., 2021; Bender et al., 2022; Wu et al., 2023b). However, this association is based mainly on studies of peripheral (serum) lactate. A metabolic flux shift toward glycolysis in brain cells results in cerebral lactate accumulation (Tobieson et al., 2019, 2022; Rasulo et al., 2020), and studies of cerebral lactate suggest a complex, dual role.

Clinical observations indicate that elevated lactate levels (hereafter “lactate” refers to cerebral lactate unless otherwise specified) are associated with persistent brain edema and neurological deficits (Tobieson et al., 2019). Lactate levels rise markedly as early as 4 h after hemorrhage and remain elevated for 48 h, with the magnitude of elevation positively correlated with the extent of brain edema (Mun-Bryce et al., 1993). Clinical correlation studies further suggest that cerebrospinal fluid lactate levels reflect the severity of brain injury, and that higher lactate levels are directly associated with an increased risk of postoperative intracranial infection and poorer clinical outcomes (Zhang et al., 2024). In solute carrier family 25 member 28 (SLC25A28) knockout mice, reduced lactate levels are accompanied by attenuated M1 polarization of microglia and decreased pro-inflammatory cytokine levels. Conversely, treatment with dimethyloxalylglycine restores lactate levels and partially reverses the anti-inflammatory effects of SLC25A28 deficiency, suggesting that elevated lactate promotes post-ICH inflammatory activation (Han et al., 2024).

Although these studies highlight the detrimental effects of elevated lactate, other evidence points in the opposite direction. Lactate has been shown to activate NF-κB signaling and upregulate vascular endothelial growth factor (VEGF), thereby promoting angiogenesis and neurogenesis (Zhou J. et al., 2018). This suggests that lactate accumulation after ICH is not merely a metabolic byproduct but also a signaling molecule that contributes to endogenous repair processes. Additional studies demonstrate that lactate promotes microglial polarization from a pro-inflammatory phenotype (M1-like) toward a reparative anti-inflammatory phenotype (M2-like) via the FOSL2–HIC1 transcriptional axis. Conversely, inhibiting lactate production with galloflavin impedes M2 polarization, supporting a requirement for lactate in maintaining an anti-inflammatory, repair-promoting microenvironment (Ai et al., 2023). Lactate has also been shown to enhance the viability of M2-polarized microglia, thereby facilitating the phagocytic clearance of the hematoma (Liu et al., 2020).

Taken together, these seemingly paradoxical findings likely represent distinct, stage-dependent roles of lactate in ICH pathophysiology rather than irreconcilable discrepancies in the literature. Specifically, studies reporting detrimental effects have predominantly focused on the acute phase or early pathophysiological processes, such as cerebral edema formation and microglial M1 polarization. By contrast, studies emphasizing beneficial effects have largely centered on later phases or reparative programs, including microglial M2 polarization, hematoma clearance, angiogenesis, and neurogenesis. Accordingly, in the acute phase after ICH, lactate accumulation is likely associated with exacerbated brain edema and inflammation. In later phases, sustained or moderately elevated lactate levels may instead favor anti-inflammatory repair and neurological recovery. This stage-dependent divergence indicates that the effects of lactate should be interpreted within the context of the specific pathophysiological time window.

## 5 Dysregulation of mitochondrial quantity and quality control

Mitochondrial quality control is generally considered to encompass mitochondrial biogenesis, mitochondrial dynamics (fusion and fission), and mitophagy, which together maintain a healthy mitochondrial pool (Archer, 2013; Suliman and Piantadosi, 2016). However, to sustain mtROS homeostasis and energy metabolism, cells require mitochondria in both quantity and quality (Ventura-Clapier et al., 2008). Accordingly, this review distinguishes insufficient mitochondrial quantity from dysregulated mitochondrial quality control and discusses them separately.

### 5.1 Insufficient mitochondrial quantity

#### 5.1.1 Inadequate compensatory mitochondrial biogenesis

Mitochondrial biogenesis is the process by which coordinated regulation of nuclear and mitochondrial genomes results in a net increase in mitochondrial content. Central to this process is peroxisome proliferator-activated receptor γ coactivator 1-α (PGC-1α), a transcriptional coactivator that enhances the activity of multiple transcription factors to drive the expression of genes involved in mitochondrial biogenesis (Kelly and Scarpulla, 2004).

Following ICH, PGC-1α is rapidly upregulated within 1 h, serving as an adaptive response to dysregulated energy metabolism and mitochondrial injury (Tang et al., 2025). Over time, PGC-1α expression continues to increase and peaks at 72 h post-hemorrhage (Tang et al., 2025), a window that closely overlaps with peak brain edema and the active phase of SBI. This temporal coincidence suggests that mitochondrial biogenesis is maximally activated in response to the most severe phase of metabolic stress following hemorrhage. However, a study of surgical brain injury—a condition with pathophysiology similar to post-ICH SBI—reported that mitochondrial transcription factor A (TFAM) is upregulated later than PGC-1α (Wang et al., 2024). As TFAM is a key downstream transcription factor of PGC-1α, this temporal lag suggests a delay between upstream signaling activation and downstream protein synthesis, thereby retarding the progression of mitochondrial biogenesis. Moreover, nuclear sirtuin 1 (SIRT1), a deacetylase of PGC-1α, is markedly reduced after ICH, increasing PGC-1α acetylation and thereby impairing its activity (Zhou Y. et al., 2018). Notably, pharmacological agents that increase PGC-1α expression have been shown to enhance mitochondrial biogenesis and improve neurological function (Zhou Y. et al., 2018; Zhang et al., 2025), indirectly supporting the notion that compensatory mitochondrial biogenesis is inadequate after ICH.

In summary, although mitochondrial biogenesis is activated as a compensatory response after ICH, this response is ultimately inadequate due to signaling delays and impaired regulator activity, compromising mitochondrial quantity and cellular energy production.

### 5.2 Dysregulation of mitochondrial quality control

#### 5.2.1 Excessive mitochondrial fission

Mitochondrial fission is primarily mediated by dynamin-related protein 1 (Drp1) (Kleele et al., 2021). Phosphorylation of Drp1 at Ser616 [p-Drp1(Ser616)] promotes Drp1 recruitment to the OMM, thereby facilitating mitochondrial fission (Kashatus et al., 2011). By contrast, phosphorylation of Drp1 at Ser637 [p-Drp1(Ser637)] suppresses mitochondrial fission (Chang and Blackstone, 2007). Studies have reported that after ICH, p-Drp1(Ser616) levels increase, whereas p-Drp1(Ser637) levels decrease (Wu et al., 2020b). In parallel, Drp1 translocates from the cytosol to mitochondria, which likewise promotes mitochondrial fission (Wu et al., 2020b).

Regarding cell-type-specific mechanisms, triggering receptor expressed on myeloid cells 1 (TREM-1) is upregulated in microglia after ICH and, via activation of the phosphoinositide 3-kinase/protein kinase B (PI3K/Akt) pathway, increases p-Drp1(Ser616) levels, thereby driving excessive microglial mitochondrial fission (An et al., 2025). Phosphoglycerate mutase family member 5 (PGAM5) is upregulated in astrocytes and promotes mitochondrial fission by reducing p-Drp1(Ser637) (He et al., 2026). Acrolein accumulates in neurons and promotes fission by driving Drp1 translocation from the cytosol to mitochondria (Wu et al., 2020a).

The endoplasmic reticulum (ER) also participates in regulating mitochondrial fission. ER–mitochondria contact sites, known as mitochondria-associated membranes (MAMs), provide platforms that facilitate Drp1-dependent fission (Friedman et al., 2011; Jiang et al., 2023). MAM formation and function depend heavily on the inositol 1,4,5-trisphosphate receptor–glucose-regulated protein 75–voltage-dependent anion channel (IP3R–GRP75–VDAC) complex (He et al., 2023). Proteomic analyses have revealed elevated succinylation of VDAC2 after ICH (Liang Y.-J. et al., 2022). These findings raise the possibility that VDAC2 alterations after ICH may perturb MAM formation and function, thereby promoting excessive mitochondrial fission, though direct evidence is currently lacking.

Overall, aberrant Drp1 activation and translocation, together with dysregulated upstream signaling, can collectively drive excessive mitochondrial fission after ICH. Excessive fission compromises mitochondrial architecture, yielding smaller, fragmented mitochondria that increase mtROS generation, reduce ATP levels, and promote apoptosis (Frank et al., 2001; Archer, 2013; Zheng et al., 2023).

Beyond Drp1, mitochondrial fission is also regulated by several additional regulators, including the Drp1-recruiting mitochondrial fission factor (Mff) (Osellame et al., 2016; Seager et al., 2024), mitochondrial dynamics proteins of 49/51 kDa (MiD49/51) (Osellame et al., 2016; Seager et al., 2024), mitochondrial fission 1 (Fis1) (Zhang and Chan, 2007), preconstriction factors such as actin and nonmuscle myosin II (NMII) (Ji W. et al., 2015; Yang and Svitkina, 2019), and the downstream scission GTPase dynamin 2 (DNM2) (Lee et al., 2016). However, current ICH research has largely centered on Drp1 expression and activity, whereas direct evidence implicating Mff, MiD49/51, Fis1, actin, NMII, and DNM2 in ICH remains scarce.

#### 5.2.2 Reduced mitochondrial fusion

Mitochondrial fusion initiates at the OMM and subsequently extends to the IMM (Song and Dorn, 2015). Fusion facilitates the exchange of matrix contents between mitochondria, thereby diluting accumulated mtDNA mutations and oxidized proteins (Archer, 2013). Moreover, it generates an interconnected mitochondrial network that enables efficient metabolite transfer and utilization, thereby enhancing ATP synthesis (Li W. et al., 2025). Key fusion mediators include mitofusins (Mfn1 and Mfn2) and optic atrophy 1 (Opa1). Mfn1 and Mfn2 localize to the OMM and primarily mediate outer membrane fusion (Chen et al., 2003), whereas Opa1 resides in the IMM and, in addition to driving inner membrane fusion, maintains cristae architecture (Frezza et al., 2006).

Following ICH, expression levels of Mfn1, Mfn2, and Opa1 are reduced (Wu et al., 2023c; Diao et al., 2024). Transmission electron microscopy has revealed marked mitochondrial ultrastructural damage, including cristae loss, cristae collapse, and reduced mitochondrial size (Wu et al., 2023c). These findings indicate that dysregulated mitochondrial quality control after ICH results from imbalanced mitochondrial dynamics, driven by both excessive fission and impaired fusion.

#### 5.2.3 Inadequate compensatory mitophagy

Appropriate mitophagy is essential for maintaining cellular homeostasis. Damaged mitochondria release excessive mtROS and pro-apoptotic factors, whereas mitophagy eliminates these dysfunctional organelles (Archer, 2013; Zorov et al., 2014). Moreover, under energy scarcity or shifting energetic demands, removal of surplus or inefficient mitochondria helps optimize the cellular energy budget (Kurihara et al., 2012).

Previous studies have shown that mitophagy-related structures and markers increase as early as 6 h and 24 h after ICH, suggesting that mitophagy is activated as a stress-induced protective response (Liu et al., 2021). The PTEN-induced kinase 1 (PINK1)/Parkin pathway is a key regulatory axis of mitophagy (Wang et al., 2023); however, in ICH models, PINK1 and Parkin protein levels decline by 3 days post-ICH (Li et al., 2021; Li J. et al., 2025). These findings indicate that, although mitophagy is triggered after ICH, dysregulation of the PINK1/Parkin pathway limits the clearance of damaged mitochondria.

Moreover, mitophagy is tightly linked to balanced mitochondrial dynamics. Under homeostatic conditions, damaged mitochondria undergo asymmetric fission, generating one healthy daughter mitochondrion that re-enters the functional mitochondrial network via fusion, whereas the other depolarized daughter mitochondrion is removed by mitophagy (Twig et al., 2008). After ICH, excessive fission coupled with reduced fusion increases the accumulation of dysfunctional mitochondria, overwhelms mitophagic clearance capacity, and thereby exacerbates cellular injury.

In summary, dysregulation of the PINK1/Parkin pathway and imbalanced mitochondrial fission–fusion dynamics after ICH jointly lead to inadequate compensatory mitophagy, thereby promoting the accumulation of damaged mitochondria and contributing to elevated mtROS, reduced ATP production, and exacerbated mitochondrial dysfunction and cellular injury.

## 6 Mitochondrial dysfunction and cell death

**FIGURE 6:**
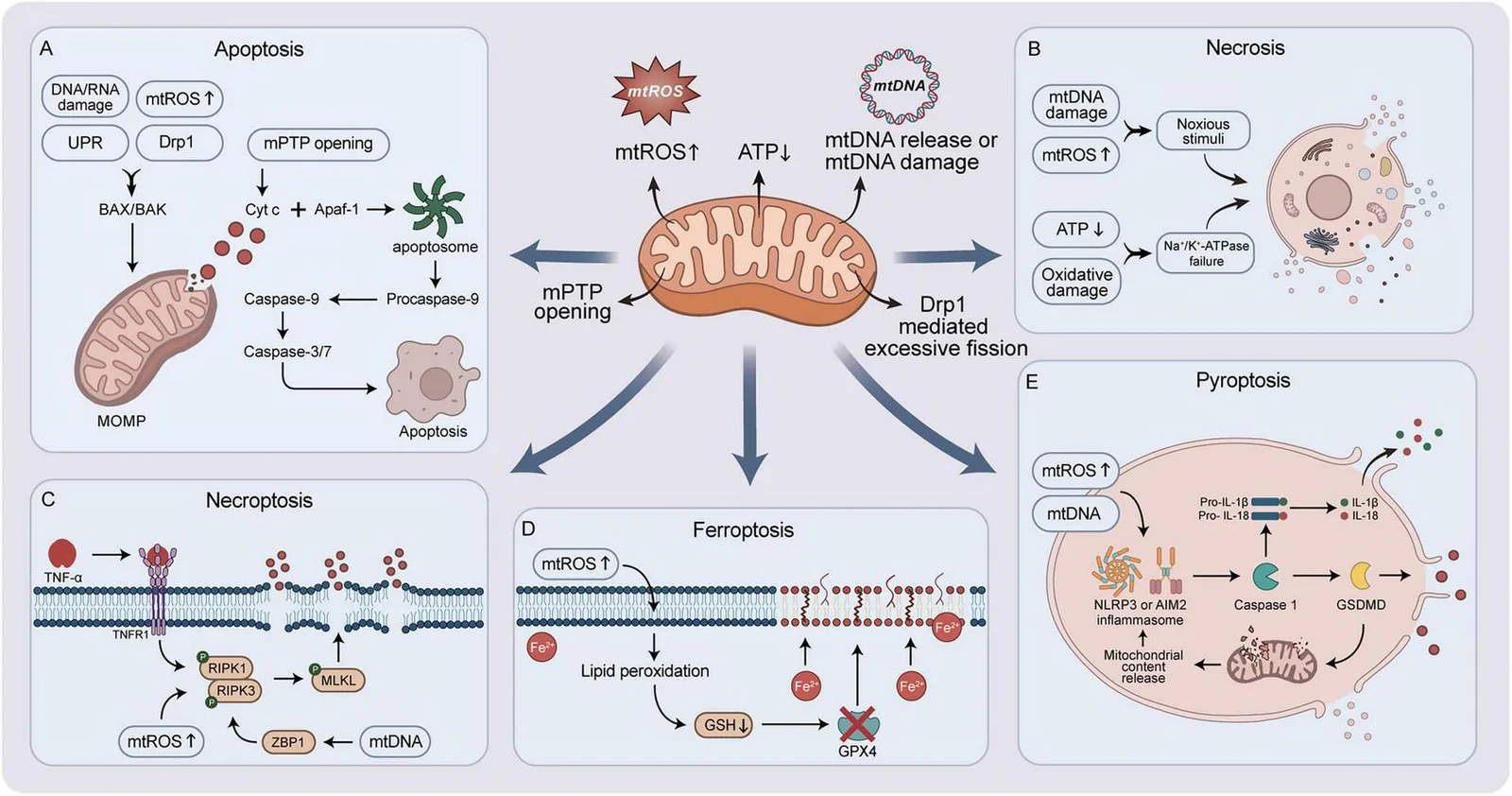
Mitochondrial dysfunction and cell death. **(A)** DNA/RNA damage, the UPR, elevated mtROS, and Drp1-mediated excessive mitochondrial fission initiate BAX/BAK-mediated MOMP. Both MOMP and sustained mPTP opening release Cyt c, which binds Apaf-1 to assemble the apoptosome, sequentially activating the caspase-9 and caspase-3/7 cascade. **(B)** Excessive mtROS and mtDNA damage act as noxious stimuli to trigger necrosis. Insufficient ATP production and oxidative damage cause Na+/K+-ATPase failure, promoting membrane rupture and necrosis. **(C)** Either TNF-α–TNFR1 signaling or elevated mtROS can initiate necroptosis. Subsequently, RIPK1 and RIPK3 assemble into the necrosome and undergo reciprocal phosphorylation. Within this complex, RIPK3 phosphorylates and activates MLKL, driving MLKL translocation to the plasma membrane, ultimately leading to necroptosis. Additionally, ZBP1 senses mtDNA released into the cytosol and activates the RIPK3–MLKL axis to promote necroptosis. **(D)** Excessive mtROS drive lipid peroxidation and deplete GSH, compromising GPX4-mediated antioxidant defense and promoting ferroptosis. **(E)** Accumulated mtROS and/or released mtDNA promote NLRP3 or AIM2 inflammasome assembly and subsequent caspase-1 activation. Caspase-1 cleaves pro-IL-1β and pro-IL-18 into mature pro-inflammatory cytokines (IL-1β and IL-18), and cleaves GSDMD to form pores in both the plasma and mitochondrial membranes. This mitochondrial disruption triggers further release of mitochondrial contents, creating a positive feedback loop that amplifies pyroptosis.

Multiple forms of cell death occur after ICH, including apoptosis, necrosis, necroptosis, ferroptosis, and pyroptosis. Some are highly pro-inflammatory and thereby exacerbate SBI. Others, although less inflammatory or even considered adaptive (Galluzzi et al., 2018), still contribute to neurological deficits and worsen SBI due to the limited regenerative capacity of neurons. Notably, mitochondrial dysfunction frequently participates in these cell death programs, either directly or indirectly (Figure 6).

### 6.1 Apoptosis

Apoptosis is an evolutionarily conserved, tightly regulated, and non-inflammatory form of cell death that proceeds through intrinsic or extrinsic pathways (Newton et al., 2024). The intrinsic pathway is initiated by intracellular stress signals, many of which stem from mitochondrial dysfunction—including DNA or RNA damage, mtROS accumulation, and the UPR (Figure 6A; Wang et al., 2015; Redza-Dutordoir and Averill-Bates, 2016; Mohammed Thangameeran et al., 2020). Frank et al. (2001) also demonstrated that Drp1-mediated excessive mitochondrial fission contributes to apoptosis initiation. Upon activation of the intrinsic apoptotic pathway, BCL2-associated X protein (BAX) and BCL2 antagonist/killer 1 (BAK) oligomerize on the OMM to form pore-like structures, triggering mitochondrial outer membrane permeabilization (MOMP) and the subsequent release of Cyt c into the cytosol. Cyt c binds Apaf-1 to assemble the apoptosome, which recruits procaspase-9 and activates it to produce active caspase-9, thereby activating caspase-3/7 and executing apoptosis (Li et al., 1997; Wei et al., 2001; Newton et al., 2024). Notably, persistent opening of the mitochondrial permeability transition pore (mPTP) after ICH can also release Cyt c, thereby engaging the intrinsic apoptotic cascade potentially independent of canonical BAX/BAK-mediated MOMP.

Although mitochondrial dysfunction does not initiate extrinsic apoptosis, it can robustly amplify or synergize with extrinsic apoptotic signaling. Extrinsic apoptosis is triggered by death ligand–receptor engagement, whereupon caspase-8 cleaves BH3-interacting domain death agonist (BID) into truncated BID, which promotes BAX/BAK activation and amplifies extrinsic signals via the mitochondrial pathway (Luo et al., 1998). This amplification loop is particularly important in type II cells (Newton et al., 2024), such as neurons, where extrinsic apoptosis is highly dependent on mitochondrial engagement and fails to proceed in its absence (Budd et al., 2000).

The apoptotic program is also finely tuned by cellular energy status. When mitochondrial damage is modest and ATP levels remain at 25–70% of normal, cells retain sufficient energy to execute apoptosis, whereas when damage is severe and ATP falls below 15%, cells instead undergo necrosis (Eguchi et al., 1997; Lieberthal et al., 1998).

### 6.2 Necrosis

Necrosis is an unregulated, passive, and accidental form of cell death triggered by severe extrinsic insults. Upon necrosis, the uncontrolled release of cellular contents—including abundant DAMPs—triggers robust inflammation and tissue injury (Newton et al., 2024).

After ICH, the mass effect of the hematoma inflicts severe mechanical injury that can directly induce necrosis (Zhang et al., 2022b). Necrosis has also been reported in the contralateral cerebral cortex, indicating that hematoma compression is not the sole cause of necrosis (Qureshi et al., 2001). Mitochondrial dysfunction leads to excessive mtROS accumulation and DNA damage. These noxious stimuli can directly trigger necrosis (Duan et al., 2016). In addition, insufficient ATP production and oxidative damage compromise Na^+^/K^+^-ATPase function, promoting cytotoxic edema and membrane rupture, ultimately leading to necrosis (Figure 6B; Lewén et al., 2000).

### 6.3 Necroptosis

Necroptosis is a regulated form of necrotic cell death that combines the highly controlled nature of apoptosis with the pro-inflammatory consequences of necrosis (Degterev et al., 2005). Binding of TNF-α to tumor necrosis factor receptor 1 (TNFR1) can initiate necroptosis when caspase-8 activity is inhibited or its regulatory control is compromised (Figure 6C; Sun et al., 2012; Chen et al., 2014). Subsequently, receptor-interacting protein kinases 1 and 3 (RIPK1 and RIPK3) assemble into the necrosome and undergo reciprocal phosphorylation. Within this complex, RIPK3 phosphorylates and activates mixed lineage kinase domain-like pseudokinase (MLKL), driving MLKL oligomerization and translocation to the plasma membrane, thereby increasing membrane permeability and compromising membrane integrity, ultimately leading to necroptosis (He et al., 2009; Sun et al., 2012; Chen et al., 2014).

Emerging evidence suggests that mitochondrial dysfunction is closely involved in necroptotic signaling. Elevated mtROS can promote RIPK1 autophosphorylation, facilitating necrosome assembly and thereby promoting necroptosis (Zhang et al., 2017; Zille et al., 2017). Additionally, mtDNA released into the cytosol can be sensed by Z-DNA binding protein 1 (ZBP1), which activates the RIPK3–MLKL axis to similarly enhance necroptosis (Figure 6C; Wang W.-Y. et al., 2022; Lai et al., 2024).

However, mitochondrial dysfunction is not an absolute prerequisite for necroptosis. Experimentally, TNF-α-induced necroptosis proceeds normally even in cells completely lacking mitochondria (Tait et al., 2013). Thus, mitochondrial dysfunction can potentiate, but is not essential for, necroptosis.

### 6.4 Ferroptosis

Ferroptosis is an iron-dependent form of regulated cell death characterized by the accumulation of lipid peroxides (Dixon et al., 2012; Kagan et al., 2017). Glutathione peroxidase 4 (GPX4) is a central regulatory enzyme of ferroptosis. It converts toxic lipid peroxides into nontoxic lipid alcohols using glutathione (GSH) as a reducing cofactor, thereby suppressing ferroptosis (Yang et al., 2014; Ursini and Maiorino, 2020).

Evidence suggests a bidirectional role for mitochondrial dysfunction in ferroptosis. On one hand, excessive mtROS drive lipid peroxidation and deplete the key reductant GSH, thereby promoting ferroptosis (Figure 6D; Gao et al., 2019; Liang D. et al., 2022). On the other hand, inhibiting mitochondrial glutaminolysis can reduce TCA cycle activity and attenuate ferroptosis (Gao et al., 2015, 2019). This raises a key question: given that mitochondrial dysfunction after ICH involves both increased mtROS and reduced TCA cycle activity, does the net effect promote or inhibit ferroptosis? Addressing this issue requires disentangling the relative contributions of these two mitochondrial alterations in ICH. Reduced TCA cycle activity, while capable of limiting ferroptosis, simultaneously diminishes the supply of reducing equivalents to the ETC, thereby decreasing mtROS generation. In ICH, however, mtROS levels do not decline despite reduced TCA cycle activity; instead, they remain persistently elevated for multiple reasons (as discussed in section “3.1 Mechanisms underlying excessive mtROS accumulation”). Under these conditions, the deleterious impact of increased mtROS outweighs the benefit conferred by reduced TCA cycle activity. Therefore, in ICH, mitochondrial dysfunction appears overall to promote ferroptosis.

### 6.5 Pyroptosis

Pyroptosis is a form of regulated cell death characterized by inflammatory cytokine release and lytic plasma membrane rupture driven by pore formation (Galluzzi et al., 2018). In response to severe insults (e.g., pathogen infection or cellular injury), danger signals promote inflammasome assembly and subsequent activation of caspase-1 (or caspase-4/5/11). Activated caspases cleave pro-interleukin-1β (pro-IL-1β) and pro-interleukin-18 (pro-IL-18) to generate the mature, bioactive cytokines IL-1β and IL-18. Caspases also cleave members of the gasdermin family, primarily gasdermin D (GSDMD), releasing an N-terminal fragment that oligomerizes and forms pores in the plasma membrane. This pore formation compromises membrane integrity and enables the release of cellular contents and inflammatory mediators, ultimately executing pyroptosis and eliciting a robust inflammatory response (Galluzzi et al., 2018; Shi et al., 2014; Ding et al., 2016).

After ICH, accumulated mtROS and/or released mtDNA can act as danger signals to promote assembly of the NLRP3 or AIM2 inflammasome (Luo et al., 2019; Gu et al., 2024), thereby triggering pyroptosis (Figure 6E). In addition, cardiolipin externalization can also contribute to NLRP3 inflammasome assembly and promote pyroptosis (Iyer et al., 2013; Elliott et al., 2018). Recent studies have shown that, beyond targeting the plasma membrane, GSDMD can also permeabilize the mitochondrial membrane by forming pores, promoting mitochondrial content release and establishing a positive-feedback amplification loop that reinforces pyroptosis (Miao et al., 2023). Thus, mitochondrial dysfunction can act upstream to initiate pyroptosis and downstream to promote and amplify its effects.

## 7 Limitations and future directions

Although a growing number of studies in recent years have underscored the central role of mitochondrial dysfunction in SBI after ICH, research in this field still faces substantial limitations. Most existing mitochondrial studies in ICH have focused primarily on the brain, whereas mitochondrial homeostasis in peripheral organs has been largely overlooked. In fact, ICH is frequently accompanied by systemic complications, including hypertension, cardiovascular events, metabolic abnormalities, and infections (Balami and Buchan, 2012; Ishiguchi et al., 2024). Therefore, an exclusive focus on mitochondrial alterations within the brain may be insufficient to fully capture the overall pathophysiological processes following ICH. Moreover, many studies based on bulk brain tissue or whole-cell lysates obscure cell-type-specific mitochondrial features, even though neurons, astrocytes, microglia, and vascular endothelial cells differ markedly in their mitochondrial metabolic profiles and vulnerability to injury (Zhang et al., 2014).

A more substantial challenge is that, although mitochondria-targeted interventions have proliferated in preclinical research, successful clinical translation remains limited. Several factors may underlie this gap. First, the pathophysiology of ICH is highly complex, and mitochondria-targeted interventions alone may therefore be insufficient to reverse the overall disease course. Second, most preclinical studies are conducted in young, healthy rodents, whereas patients encountered in clinical practice are typically older and often present with multiple comorbidities. Third, some pharmacological interventions targeting dysfunctional mitochondria may also affect healthy mitochondria, as well as cause other significant adverse effects. For example, some compounds reduce mtROS yet concurrently suppress mitochondrial ATP production (Fink et al., 2012; Reily et al., 2013). In addition, certain drug-delivery systems or viral vectors can provoke robust immune responses (Dobrovolskaia and McNeil, 2007; Mingozzi and High, 2013). Finally, the BBB and the narrow therapeutic window after ICH make it challenging for drugs to penetrate the brain and exert therapeutic effects in a timely manner.

Future studies should therefore address these bottlenecks from both mechanistic and translational perspectives. At the mechanistic level, studies should elucidate not only mitochondrial dysfunction but also protective mechanisms, such as increased mitochondrial one-carbon metabolic flux and astrocyte-to-neuron mitochondrial transfer (Tashiro et al., 2022; Liu et al., 2024c). In addition, spatial transcriptomics and single-cell multi-omics should be leveraged to delineate how mitochondrial function differs across disease stages and cell types after ICH, and to extend the investigation beyond the brain to peripheral tissues and cell populations.

At the translational level, future drug-delivery strategies should evolve from simple brain penetration to lesion targeting, and from cellular uptake to mitochondrial delivery, thereby enabling a lesion-cell-mitochondrion-oriented, multi-tier targeted delivery system. Encouragingly, recent advances in nanotechnology have provided new avenues for brain-targeted strategies. Nanocarriers, including polymeric nanoparticles, lipid-based nanostructures, inorganic scaffolds, and biomimetic or hybrid nanoplatforms, possess highly tunable physicochemical properties and can be structurally engineered to achieve sustained release, targeted delivery, and stimulus-responsive functions (Liao et al., 2026). Among these platforms, CeO_2_ nanoenzymes have shown considerable promise for treating mitochondrial dysfunction. Through reversible Ce^3+^/Ce^4+^ redox cycling, they can mimic antioxidant enzymes such as superoxide dismutase and catalase, scavenge excessive mtROS, restore mitochondrial membrane potential, and improve ATP levels (Liao et al., 2024, 2025b). The construction of multi-tier targeted nanosystems may also draw on the work of Liao et al. (2024, 2025b) in ischemic stroke, in which superparamagnetic iron oxide nanoparticles combined with an external magnetic field were used to guide brain-region targeting, BV2 microglial cell membrane coating enhanced BBB penetration and inflammatory lesion homing, and triphenylphosphine further enabled intracellular mitochondrial targeting. Correspondingly, key evaluation endpoints should be developed in parallel with these targeting strategies, including lesion enrichment ratios, intramitochondrial drug exposure, and normalization of relevant mitochondrial functional readouts. These endpoints may improve the precision of therapeutic evaluation and facilitate the clinical translation of mitochondria-targeted interventions.

## 8 Conclusion

Mitochondrial dysfunction in ICH is a multidimensional pathological process involving excessive accumulation of mtROS, dysregulation of energy metabolism, and dysregulation of mitochondrial quantity and quality control. These dysfunctions do not occur in isolation; rather, they crosstalk with one another, ultimately amplifying oxidative stress, inflammation, BBB disruption, and energy crisis, and driving multiple forms of cell death (Figures 2, 6). Consequently, mitochondrial dysfunction acts not only as a consequence of SBI but also as a key driver of its exacerbation.

By analyzing and synthesizing the molecular mechanisms and pathological consequences of mitochondrial dysfunction, this review facilitates a deeper understanding of the complex pathophysiological cascades underlying SBI after ICH. These insights also provide valuable mechanistic insights for other conditions characterized by similar mitochondrial pathophysiological features, including neurodegenerative disorders, cardiovascular and renal diseases, and cancers (Tang C. et al., 2021; Xu et al., 2025).

Future research should deepen our understanding of mitochondrial biology and develop more rational and precise targeted interventions to alleviate SBI and improve outcomes in ICH patients.
